# Molecular and Photosynthetic Responses to Prolonged Darkness and Subsequent Acclimation to Re-Illumination in the Diatom *Phaeodactylum tricornutum*

**DOI:** 10.1371/journal.pone.0058722

**Published:** 2013-03-08

**Authors:** Marianne Nymark, Kristin C. Valle, Kasper Hancke, Per Winge, Kjersti Andresen, Geir Johnsen, Atle M. Bones, Tore Brembu

**Affiliations:** 1 Department of Biology, Norwegian University of Science and Technology, Trondheim, Norway; University of Hyderabad, India

## Abstract

Photosynthetic diatoms that live suspended throughout the water column will constantly be swept up and down by vertical mixing. When returned to the photic zone after experiencing longer periods in darkness, mechanisms exist that enable the diatoms both to survive sudden light exposure and immediately utilize the available energy in photosynthesis and growth. We have investigated both the response to prolonged darkness and the re-acclimation to moderate intensity white irradiance (E = 100 µmol m^−2^ s^−1^) in the diatom *Phaeodactylum tricornutum*, using an integrated approach involving global transcriptional profiling, pigment analyses, imaging and photo-physiological measurements. The responses were studied during continuous white light, after 48 h of dark treatment and after 0.5 h, 6 h, and 24 h of re-exposure to the initial irradiance. The analyses resulted in several intriguing findings. Dark treatment of the cells led to 1) significantly decreased nuclear transcriptional activity, 2) distinct intracellular changes, 3) fixed ratios of the light-harvesting pigments despite a decrease in the total cell pigment pool, and 4) only a minor drop in photosynthetic efficiency (Φ_PSII_max_). Re-introduction of the cells to the initial light conditions revealed 5) distinct expression profiles for nuclear genes involved in photosynthesis and those involved in photoprotection, 6) rapid rise in photosynthetic parameters (α and rETR_max_) within 0.5 h of re-exposure to light despite a very modest *de novo* synthesis of photosynthetic compounds, and 7) increasingly efficient resonance energy transfer from fucoxanthin chlorophyll *a*/*c*-binding protein complexes to photosystem II reaction centers during the first 0.5 h, supporting the observations stated in 6). In summary, the results show that despite extensive transcriptional, metabolic and intracellular changes, the ability of cells to perform photosynthesis was kept intact during the length of the experiment. We conclude that *P. tricornutum* maintains a functional photosynthetic apparatus during dark periods that enables prompt recovery upon re-illumination.

## Introduction

Diatoms (Bacillariophyceae) constitute a species-rich group of unicellular, photosynthetic eukaryotes found worldwide in freshwater and ocean systems. They are an ecologically important group of phytoplankton, being responsible for approximately 40% of marine primary productivity [1], and function as a primary source of food for many aquatic animals. Since diatoms are passively transported by currents and turbulent mixing, they need to be able to cope with light potentially strong enough to cause photodamage at the sea surface, as well as being carried to depths below the photic zone where solar energy is too low to fuel oxygenic photosynthesis [2], [3]. The main pigments responsible for light harvesting in diatoms differ considerably from those in green algae and higher plants, resulting in very different absorption capabilities [4]. This has a major influence on photosynthetic performance in a rapidly changing light environment. The main light-harvesting pigments in diatoms include chlorophylls (Chl) *a* and *c* and the carotenoid fucoxanthin (Fuco), which are bound to fucoxanthin Chl *a*/*c*-binding proteins [4], [5]. The antenna proteins form a scaffold that facilitates the coordinated positioning of these light-harvesting pigments in relation to each other [6], [7]. The diatom pigment protein complexes are called fucoxanthin-chlorophyll protein (FCP) complexes; the latest model of the FCP pigment stoichiometry suggests each FCP monomer binds eight molecules of Chl *a*, eight Fuco and two Chl *c* molecules in its protein backbone [6]. These monomers are further organized into trimers and oligomers in the peripheral antennae of PSII [8]. The subunits of the special PSI antenna complex have recently also been found to contain the pigmentation specific for PSII FCP monomers, exhibiting unchanged Chl *a*:Chl *c*:Fuco during high light (HL) and low light (LL) exposure [9].

The main pigments involved in photoprotection are the xanthophyll cycle pigments diadinoxanthin (Diadino) and diatoxanthin (Diato; [10]). The FCP monomer has also recently been suggested to contain at least one protein-bound molecule of Diadino [11]. The remaining Diadino cycle pigments appear to be dissolved in a lipid shield surrounding the FCPs, where they can readily be de-epoxidized to Diato in HL [11]. These unbound, lipid-dissolved Diadino cycle pigments are thought to function as antioxidants and/or as a reservoir for the synthesis of the light-harvesting pigment Fuco after a shift from HL to LL [12].

Several diatom species have been found to survive weeks to months [13]–[15] and in some cases years [13]–[15] in complete darkness as vegetative resting cells. The resting cells are externally similar to normally growing vegetative cells, but show physiological and internal structural modifications. Common traits of diatom resting cells are low metabolic rates and condensed cytoplasm with chloroplasts localized to the cell center. Within hours to days after being returned to favorable growth conditions, normal internal cell structure is regained and cell division resumes [13]–[18]. The time from re-illumination to start of exponential growth is dependent on species and duration of the dark period [16], [17].

*Phaeodactylum tricornutum* has been reported to survive up to six months in complete darkness [19]. In *P. tricornutum* cultures exposed to a prolonged period of darkness, cell division ceases and cells are arrested in the G1 phase [20], [21]. When G1-arrested *P. tricornutum* cells were subjected to light after a 20 h dark period, chloroplast division started 5 h after re-illumination and cell division was found to take place 8–12 h after transfer from darkness to light [21]. Griffiths [22] reported that the photosynthetic capacity (the maximum rate of carbon fixation during photosynthesis), measured in terms of ^14^CO_2_ assimilation in *P. tricornutum* cultures, varied greatly depending on growth conditions, but in general remained high during a prolonged period of darkness. He observed no major changes in the Chl *a* concentration or total protein content during the dark period. In some cases, dark treatment of the cells was found to be beneficial because prolonged periods of growth in light-dark cycles reduced the photosynthetic capacity of the cells. It was suggested that the changes in photosynthetic capacity observed under different conditions are caused by changes in the activity of proteins taking part in the photosynthetic machinery.

Knowledge about the underlying molecular mechanisms during shifts between periods of prolonged darkness and light is limited. Leblanc and co-workers [23] examined for the first time the expression of a gene encoding a fucoxanthin-chlorophyll *a*/*c*-binding protein in dark-acclimated cell cultures of the diatom *Thalassiosira weissflogii*. After a prolonged period of darkness, the expression of this gene stabilized at a constant low level. Re-illumination caused increasing mRNA levels, which peaked after 6–8 h. Expression profiling of diatom genes involved in synthesis of carotenoids in response to light of different quality and intensity after dark treatment has been performed by Coesel and co-workers [24]. White light of moderate intensity (E = 175 µmol m^−2^ s^−1^) caused a rise in expression levels of genes encoding carotenoid biosynthesis enzymes; most genes peaked after 5–8 h of light exposure. Expression profiles of several genes encoding cell cycle regulating proteins from dark-treated cultures and cultures re-exposed to light after dark treatment have also been studied [21]. Nevertheless, the molecular mechanisms behind acclimation to light after a prolonged dark period are still poorly understood.

The existing knowledge on dark-light transitions in diatoms is fragmented and often restrained to one biological level and/or single method analyses. In this study we aim to present a comprehensive overview of multilevel cellular events associated to prolonged dark treatment and subsequent re-acclimation to light in the marine diatom *P. tricornutum*.

## Results

*P. tricornutum* cultures grown under continuous white light (CWL) were transferred to complete darkness for 48 h (D48) before they were re-exposed to the initial white light (WL) for 0.5 h, 6 h and 24 h. Material was harvested from all five treatments. The global gene expression status, pigment concentrations, photosynthetic parameters, *in vivo* pigment resonance energy transfer efficiency and intracellular structure were examined in the harvested material.

### Global Messenger RNA Changes

**Figure 1:**
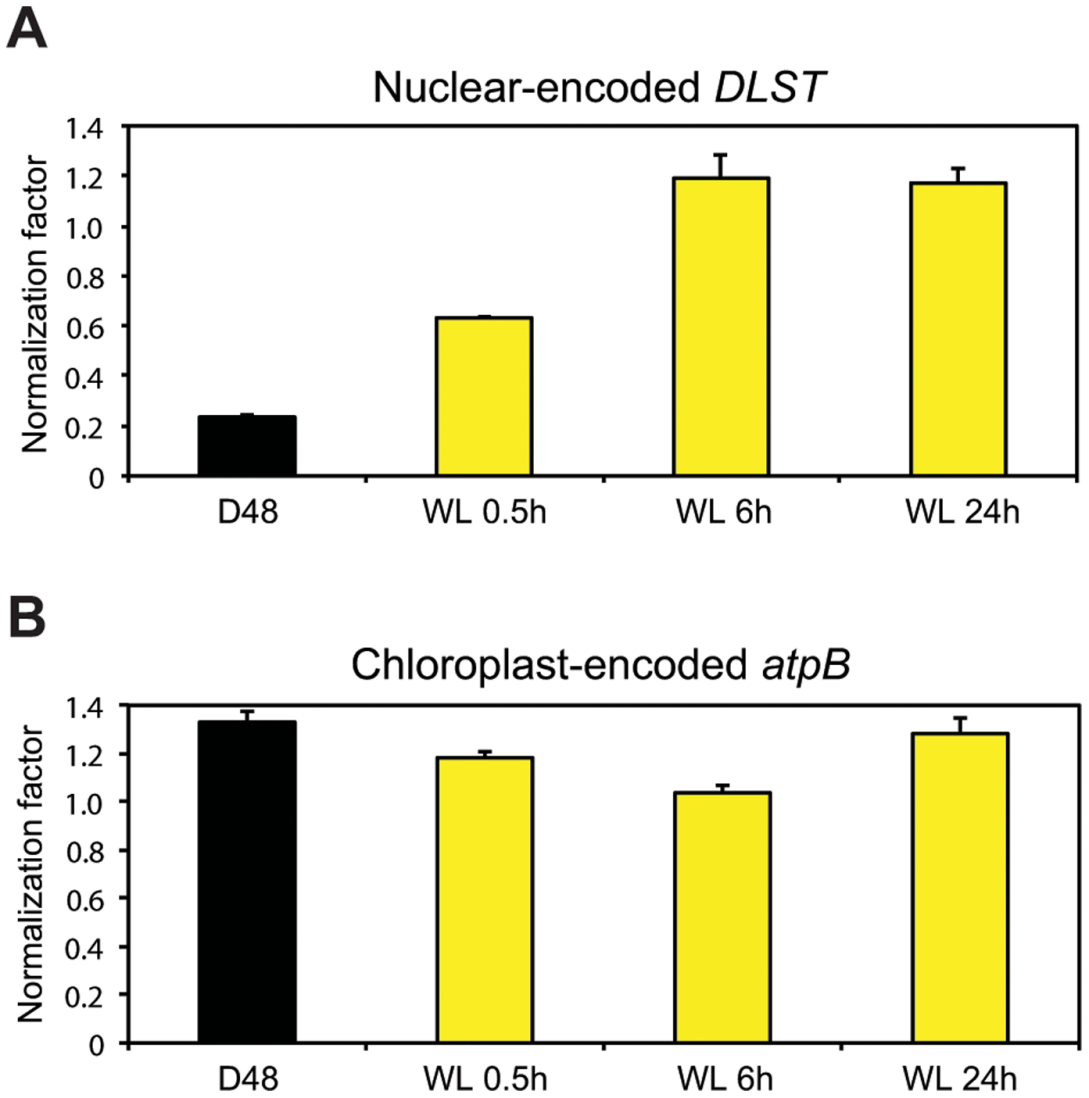
Nuclear and chloroplastic transcription is differentially regulated during prolonged darkness. qRT-PCR normalization factors were estimated by comparing the levels of the nuclear-encoded *DLST* transcript (A) or the chloroplast-encoded *atpB* transcript (B) in three biological replicas of dark-treated cells (D48) and cells re-exposed to WL for 0.5, 6 and 24 h to the levels found in CWL cells. Equal amounts of total RNA was used as starting material for the cDNA synthesis. Bars represent the average *DLST* or *atpB* normalization factor as calculated by REST2009 from two (A) or three (B) different qRT-PCR runs.

**Figure 2:**
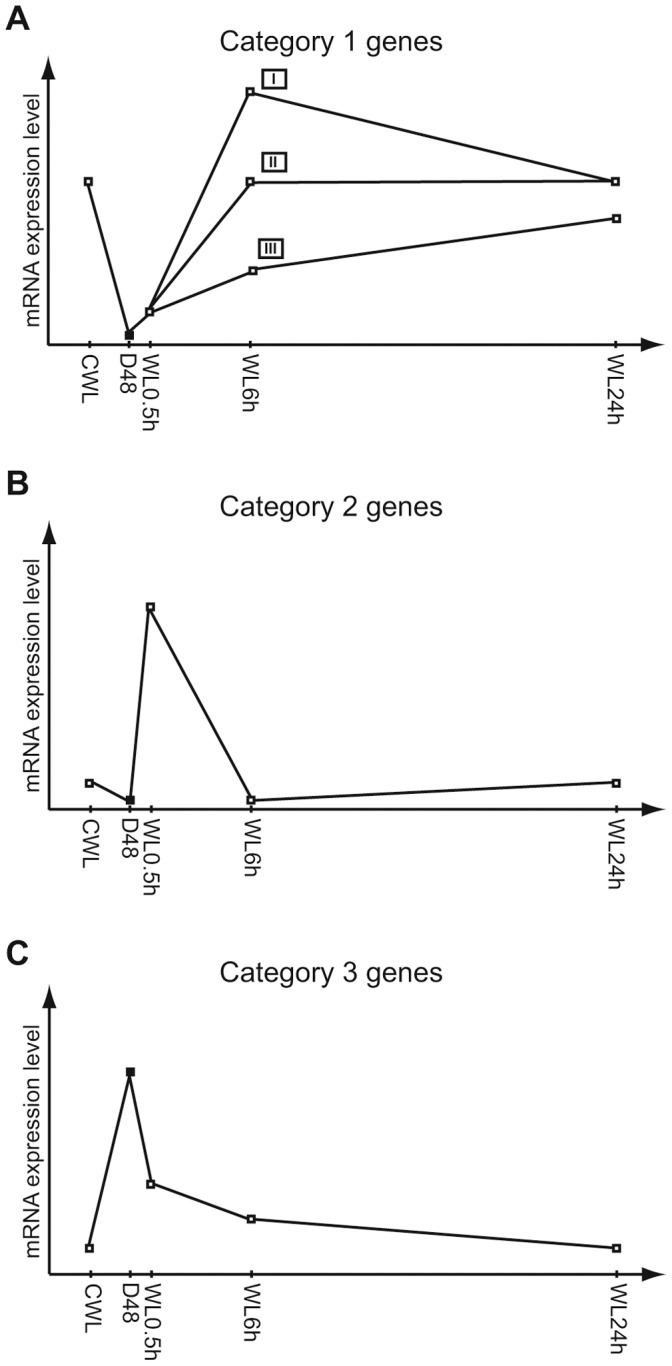
Schematic gene expression profiles of photosynthesis-associated nuclear genes and photoreceptors. Expression patterns describing the mRNA expression levels at continuous white light (CWL), after 48 h dark treatment (D48) and re-exposure to white light (WL) for 0.5, 6, and 24 h. A) Light-responsive expression patterns common for category 1 genes representing the majority of the PhANGs characterized by low transcriptional activity in darkness, modest induction by 0.5 h re-exposure to WL and generally high expression when exposed to WL for longer periods of time. I: Gene expression peaking after 6 h of re-exposure to WL. II: Gene expression stabilizing at CWL level after only 6 h of re-exposure to WL. III: Gene expression steadily increasing during 24 h of re-exposure to WL, but never reaching CWL levels. B) Expression pattern representative for category 2 genes including several genes belonging to the *LHC* family encoding proteins believed to have a photoprotective function, as well as for the *POR4* gene and two photoreceptor genes. Genes are characterized by being strongly, but transiently induced by WL exposure for 0.5 h. C) Expression pattern representative for the category 3 genes including a few PhANGs and a photoreceptor gene characterized by high transcriptional activity in darkness and a gradual reduction towards CWL levels during 24 h of WL re-exposure.

Messenger RNA (mRNA) constitutes 1–5% of total cellular RNA, depending on organism, cell type and physiological state [25]. Total RNA served as starting material for both microarray and quantitative real-time PCR (qRT-PCR) analyses. During preparations of samples for the microarray analysis, complementary RNA (cRNA) was produced from the polyadenylated RNA portion of the total RNA. The amounts of incorporated Cy3 dye in the cRNA in each sample are measured and equal amounts of cRNA are hybridized to the individual arrays. When performing qRT-PCR analyses, however, equal amounts of total RNA functions as template for complementary DNA (cDNA) synthesis. As a result, unequal amounts of cDNA might be produced and compared during the qRT-PCR analysis if the percentage of mRNA present in the total RNA differs between samples that have been exposed to different treatments. In the microarray analysis, the *DLST* gene (Phatr2_45557) was found to be unaffected by the dark-light treatment, and was therefore chosen as a reference gene for normalization of qRT-PCR expression data from nuclear-encoded genes. The normalization factor was close to 1 in the qRT-PCR analysis on material harvested after 6 and 24 h re-exposure to light (Figure 1A), as was expected based on the microarray results. However, it was much lower than 1 for the D48 samples (approx. 0.23) and WL 0.5 h (approx. 0.63). The achieved normalization factors indicate that the nuclear-encoded mRNA portion of total cellular RNA was lower in the D48 samples and to a certain degree also in the WL 0.5 h, compared to CWL. To investigate whether the total mRNA pool truly varied during the experiment, poly(A) mRNA was isolated from equal amounts of total RNA from all samples. There was significantly less poly(A) mRNA in the D48 samples (approximately 30%) and samples re-exposed to WL for 0.5 h (approximately 60%) compared to CWL samples (Table 1). No significant differences were found between the mRNA pool in CWL cultures and cultures re-exposed to WL for 6 and 24 h. These results are supported by the expression profiles for many photosynthesis-associated nuclear genes (PhANGs) described below (Figure 2A). Large amounts of mRNA were transcribed from these genes when the cells were exposed to longer periods of light. In contrast, very low transcript levels were detected for the same genes when analyzing material from D48 cultures. Such global mRNA changes might affect the expression ratios estimated by microarray analyses for nuclear-encoded genes [26], [27], possibly leading to a slight underestimation of down-regulated genes and a slight overestimation of up-regulated genes when comparing D48 samples to CWL samples. The D48-treatment did not appear to have the same effect on transcriptional activity in the chloroplast as in the nucleus. The chloroplast genes were found to be highly expressed at all times during the experiment (all Ct values ≤22), and the qRT-PCR normalization factor for the chloroplast-encoded reference gene was close to 1 for all treatments (Figure 1B).

**Table 1:** mRNA recovery from total RNA.

Sample ID	Average mRNA recovery from 4.5 µgtotal RNA (ng)	SD	P-value (Student’s t-test)	Percentage of the CWL mRNA pool
CWL	36,8	4,4		100
D	11,5^a^	3,1	0,01	31,4
WL 0.5 h	23,7	2,7	0,03	64,3
WL 6 h	33,3	5,4	0,19	90,5
WL 24 h	39,6	0,9	0,37	107,7

Polyadenylated mRNA isolated from total RNA from three biological replicates of cells grown under continuous white light (CWL), 48 h dark-treated cells (D48) and cells re-exposed to white light (WL) for 0.5, 6 and 24 h. Equal amounts of total RNA was used as starting material.

a The mRNA concentration for one of the biological replicates for the dark-treated cultures were <0.20 ng/µl (<8 ng in total), which is out of range for the Qubit fluorometer. The value for this replicate was therefore set to 8 ng in the further calculations.

### Transcriptional Profiling of Nuclear and Chloroplast Transcripts

Transcriptional profiles from D48 cells and WL 0.5–24 h cells were compared to the CWL cell profile. A large number of genes were affected by the transition from light to dark and subsequent re-exposure to the initial irradiance. Approximately 70% of the probes on the microarray were significantly regulated when comparing D48 samples and WL 0.5 h with CWL samples. The differences decreased with light exposure time; however, 24 h after re-exposure to the initial irradiance, 17% of the probes were still differentially regulated. In this work we have chosen to focus on a subset of photosynthesis-associated genes and genes encoding putative photoreceptors.

The transcriptional analyses revealed that the majority of the photosynthesis-associated nuclear genes (PhANGs) and a few photoreceptor genes could be divided into three main categories based on their gene expression profiles. 1) Category 1 includes genes that were highly expressed in CWL, displayed a low expression in darkness, were modestly induced by 0.5 h re-exposure to WL and increased towards CWL expression level during the following hours (WL 6 and 24 h). The expression profiles representing category 1 genes are schematically drawn in Figure 2AI–III. 2) Category 2 describes genes that were strongly, but transiently induced by moderate light intensity and peaked after 0.5 h of re-exposure to light. In darkness and when exposed to longer periods of light these genes showed a low to moderate expression. The expression profile representing category 2 genes are schematically drawn in Figure 2B. 3) Category 3 represents genes that showed highest expression after prolonged darkness, followed by a gradual reduction in transcript levels toward CWL levels during the next hours of re-illumination. The expression profile representing category 3 genes are schematically drawn in Figure 2C.

#### Synthesis of chlorophyll *a*

**Figure 3:**
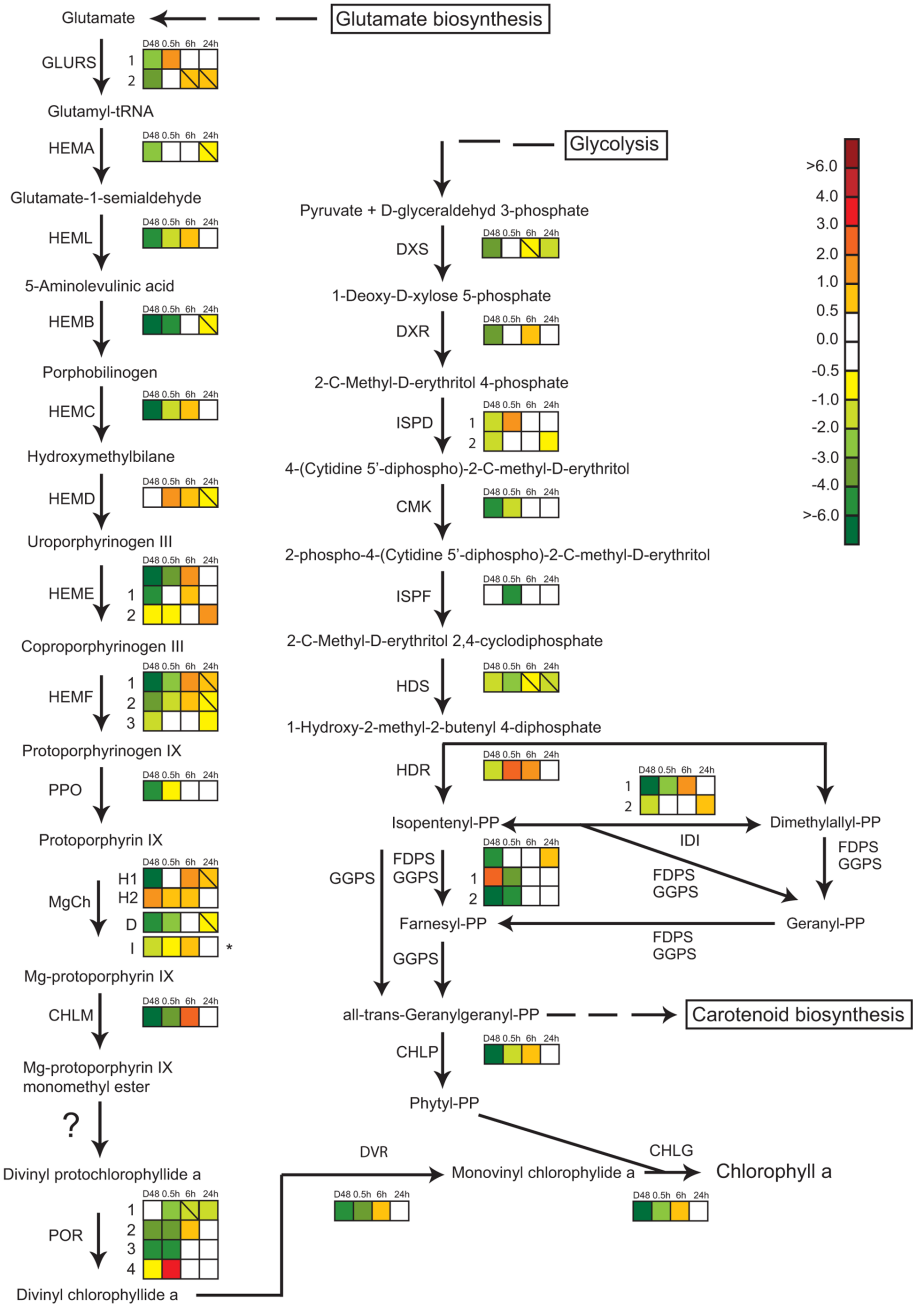
Gene expression responses in the hypothesized chlorophyll a and non-mevalonate pathways in *P. tricornutum*. Colored squares indicate the regulation pattern of genes encoding putative enzymes functioning in the two pathways after dark treatment for 48 h (D48) and re-exposure to white light (WL) for 0.5, 6 and 24 h. Continous white light (CWL) were control conditions. Squares with a diagonal line inside indicate genes with an expression ratio greater than +/−0.5 that are not significantly regulated. The asterisk marking the expression pattern of subunit I of Mg-chelatase (MgCh) indicates that the gene is chloroplast encoded, and that the expression ratios was assessed by qRT-PCR instead of microarray analysis. The scale on the right represents gene expression ratio values, log_2_ transformed. The gene encoding Mg-protoporphyrin IX monomethyl ester cyclase responsible for converting Mg-protoporphyrin IX monomethyl ester to divinyl protochlorophyllide *a* in higher plants [108] is absent in *P. tricornutum*, and this step is marked with a question mark in the figure. The abbreviations used are: GLURS: glutamyl-tRNA synthetase; HEMA: glutamyl-tRNA reductase; HEML: glutamate-1-semialdehyde 2,1-aminomutase, HEMB: porphobilinogen synthase; HEMC: hydroxymethylbilane synthase; HEMD: uroporphyrinogen-III synthase; HEME: uroporphyrinogen decarboxylase; HEMF: coproporphyrinogen III oxidase; PPO: protoporphyrinogen oxidase; MgCh: magnesium chelatase (comprising of subunits H, D and I); CHLM: Mg-protoporphyrin IX methyl transferase; POR: protochlorophyllide oxidoreductase; DVR: divinyl protochlorophyllide a 8-vinyl reductase; CHLG: chlorophyll synthase; DXS: deoxyxylulose-5-phosphate synthase; DXR: 1-deoxy-D-xylulose 5-phosphate reductoisomerase; ISPD: 2-C-methyl-D-erythritol 4-phosphate cytidylyltransferase; CMK: 4-diphosphocytidyl-2-C-methyl-D-erythritol kinase; ISPF: 2-C-methyl-D-erythritol 2,4-cyclodiphosphate synthase; HDS: 1-hydroxy-2-methyl-2-(E)-butenyl 4-diphosphate synthase; HDR: 4-hydroxy-3-methylbut-2-enyl diphosphate reductase; FDPS: farnesyl diphosphate synthase; GGPS: geranylgeranyl pyrophosphate synthase; IDI: isopentenyl pyrophosphate:dimethylallyl pyrophosphate isomerise; CHLP: geranylgeranyl reductase.

The multistep Chl *a* pathway of *P. tricornutum* described in Nymark et al. [28] involves the action of enzymes encoded by both single genes and multi-gene families, all but one being encoded by nuclear genes. Chlorophyll synthase, which catalyzes the final step of the pathway, uses monovinyl chlorophyllide and the terpenoid phytyl diphosphate as substrates to form Chl *a* [29]. The formation of phytyl diphosphate involves the non-mevalonate pathway, which operates in chloroplasts [30]. D48-treatment resulted in transcriptional down-regulation of all genes encoding enzymes of the two pathways except three (*HEMD*, *CHLH_2*, *POR1*) from the Chl *a* pathway and two genes (*ISPF*, *GGPS_1*) involved in terpenoid synthesis (Figure 3). After 24 h of re-exposure to the initial irradiance, practically no differences from the CWL cells could be observed. Category 1 expression profiles (Figure 2A) describes the most common expression patterns of genes in the Chl *a* pathway and the latest steps before formation of phytyl diphosphate. These genes displayed high expression in CWL cells, very low expression in D48 cells and a small rise in expression level in WL 0.5 h cells. After 6 h of WL several genes reached an expression peak (Figure 2AI), while others were close to the CWL expression levels (Figure 2AII). 24 h after onset of light, the expression levels seemed to stabilize at the CWL levels. The *POR4* and *GLURS_1* genes showed an atypical response to the re-illumination compared to the other genes in the Chl *a* pathway. These two genes are generally recognized by low to moderate transcript levels, but showed a strong light induction and peaked after 0.5 h of WL exposure fitting the category 2 expression profile (Figure 2B). The transcript from the *CHLH_2* gene was found to be most abundant in darkness and displayed a gradual decline towards CWL levels after re-illumination representing the category 3 expression profile (Figure 2C).

#### Synthesis of carotenoids

Light-harvesting and photo-protective pigments in diatoms are produced through the carotenoid biosynthetic pathway. Recent research has led to the identification of genes encoding enzymes responsible for the initial steps of this pathway leading to β-carotene [31]. The intermediate products between β-carotene and the end products Diadino and Fuco are also identified [31], but the genes that encode the enzymes functioning in the last part of the pathway are still unknown. As for most genes involved in the synthesis of Chl *a*, most of the carotenoid genes were expressed at lower levels in darkness than in light, and a 24 h re-exposure to WL brought the transcription levels close or back to CWL levels (Figure S1). The majority of the carotenoid synthesis genes displayed lower general expression levels in light than most of the genes encoding enzymes needed for Chl *a* formation. The expression patterns of the gene encoding lycopene beta cyclase (LCYB), as well as two of the gene candidates encoding zeaxanthin epoxidase (ZEP1 and ZEP2), were similar to what is described in Figure 2AI–II and could be classified as category 1 genes. The transcription of *LCYB* deviated somewhat from the pattern depicted in Figure 2AI by not responding to the 0.5 h WL exposure after D48-treatment. The expression profiles of *ζ-carotene desaturase* (*ZDS*) and *ZEP3* are comparable to the category 2 expression pattern (Figure 2B), but in contrast to the presented pattern the *ZDS* and *ZEP3* genes are somewhat higher expressed in darkness than in CWL.

#### Light-harvesting complex proteins

**Figure 4:**
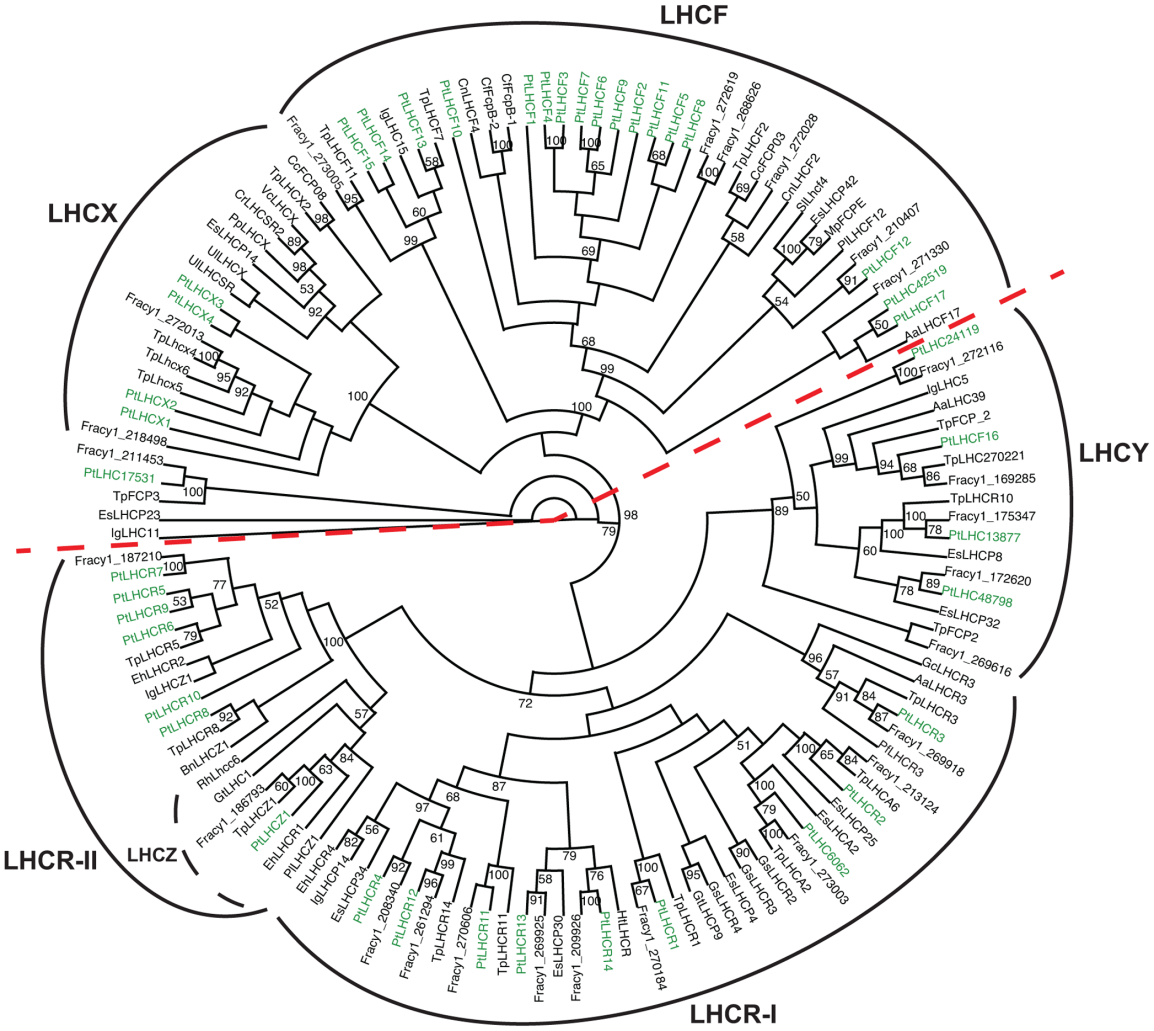
Cladogram of the antenna proteins of *P. tricornutum*. Phylogenetic analysis of all predicted *P. tricornutum* antenna proteins belonging to the light-harvesting complex (LHC) superfamily, together with other Stramenopile LHC proteins sequences as well as sequences from cryptophytes, haptophytes, rhodophytes, viridiplantae, rhizaria and an alveolate. LHCs from *P. tricornutum* are indicated in green. The two main clades are separated by a dashed red line. The abbreviations used are Aa: *Aureococcus anophagefferens*; Bn: *Bigelowiella natans*; Cc: *Cyclotella cryptica*; Cf: *Cylindrotheca fusiformis*; Cn: *Chaetoceros neogracile*; Cr: *Chl amydomonas reinhardtii*; Eh: *Emiliania huxleyi*; Es: *Ectocarpus siliculosus*; Fracy1: *Fragilariopsis cylindrus*; Gc: *Gracilaria changii*; Gs: *Galdieria sulphuraria;* Gt: *Guillardia theta*; Ht: *Heterocapsa triquetra*; Ig: *Isochrysis galbana*; Mp: *Macrocystis pyrifera*; Sl: *Saccharina latissima*; Pf: *Pseudochattonella farcimen*; Pl: *Pavlova lutheri*; Pp: *Physcomitrella patens subsp. Patens*; Pt: *Phaeodactylum tricornutum*; Rh: *Rhodomonas sp. CS24*; Tp: *Thalassiosira pseudonana*; Ul = *Ulva linza;* Vc: *Volvox carteri f. nagariensis*. The accession numbers corresponding to the protein sequences used in the analysis are listed in Table S3.

A phylogenetic analysis was performed for all predicted *P. tricornutum* antenna proteins belonging to the light-harvesting complex (LHC) superfamily, together with other Stramenopile (Heterokont) LHC proteins sequences as well as sequences from cryptophytes, haptophytes, rhodophytes, viridiplantae, rhizaria and an alveolate. The resulting cladogram is presented in Figure 4. 35 out of 42 predicted light-harvesting complex genes have previously been annotated and divided into three main groups [32], [33] the *LHCF*s, encoding the major fucoxanthin Chl *a*/*c* proteins, the red algal-like *LHCR*s and the stress-responsive LI818/LHCSR-like *LHCX*s. In our cladogram, two main groups, each divided into two subgroups, can be clearly distinguished and have high bootstrap support (Figure 4). The first group contains the LHCF subgroup, including all but one of the *P. tricornutum* LHCFs, and the LHCX subgroup. The second group comprises a new subgroup named LHCY, including LHCF16 and three formerly unclassified *P. tricornutum* LHC proteins (LHC24119, LHC48798, LHC13877; numbers refer to protein IDs in the JGI Phatr2 database) and the LHCR subgroup. Interestingly, the phylogenetic analyses reveal a further partitioning of the LHCR subgroup. In our cladogram, the LHCR5-10 proteins (LHCR-II) form a clade separate from the major LHCR group (LHCR1-4, 11–14; LHCR-I), together with a group of LHC proteins with unknown function called LHCZs [34]. An LHCZ protein was also identified in *P. tricornutum* and named LHCZ1 (Phatr2_15820). The phylogenetic analysis of the LHC proteins also suggests that the unclassified LHC protein LHC6062 should be considered as a member of the LHCR-I group and the LHC42519 protein as an LHCF. A deviant *P. tricornutum* LHC protein called LHC17531 is found as part of an outgroup in the cladogram. In addition to the LHC proteins, *P. tricornutum* also express a member of the extended LHC superfamily named LHL1 that has recently been classified as a red lineage chlorophyll *a/b*-binding (CAB)-like protein (RedCAP) [35]. The LHL1 protein sequence was not included in the phylogenetic analyses of the LHC proteins due to low sequence similarities outside of the two chlorophyll-binding motifs [36].

**Figure 5:**
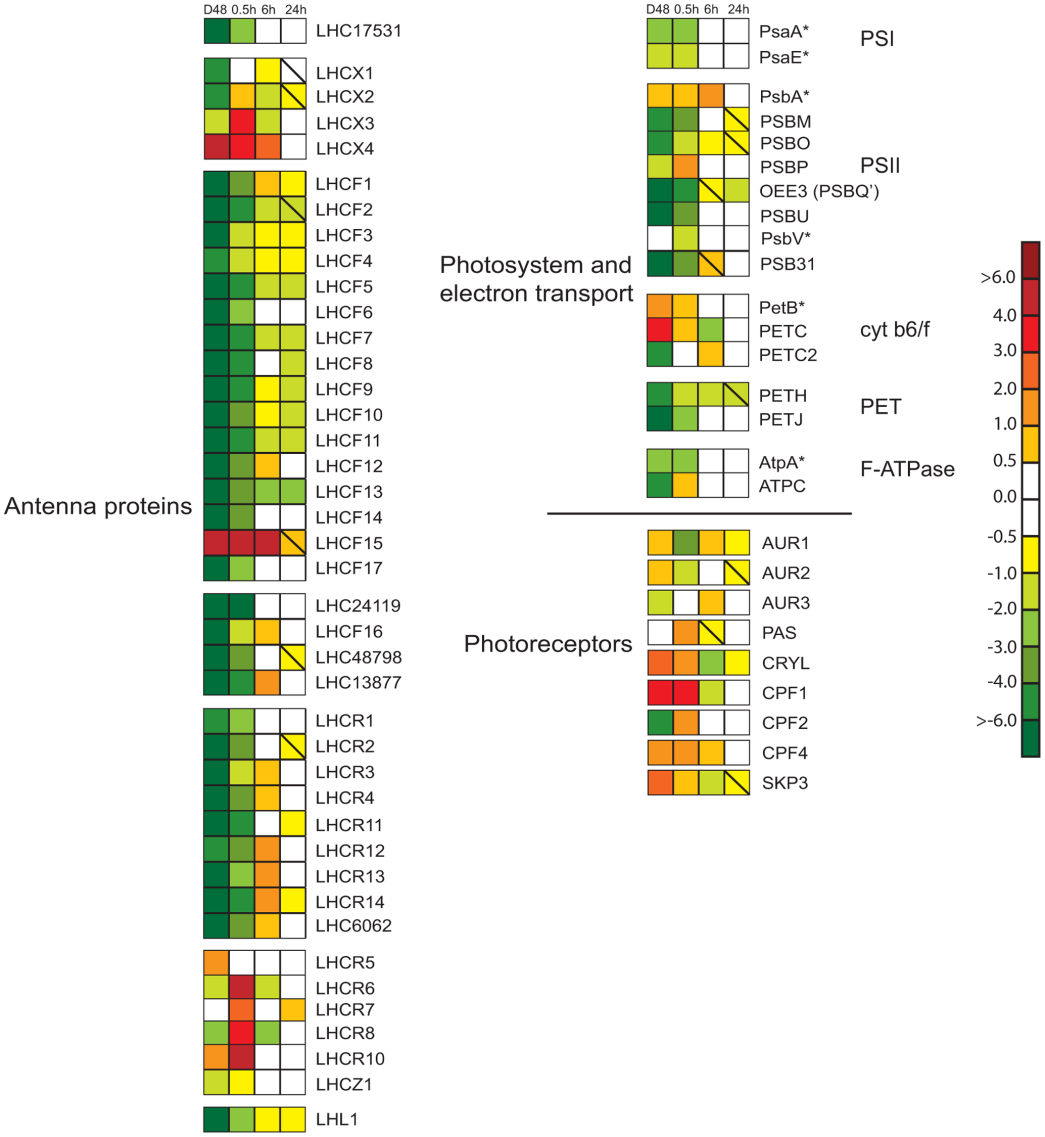
Expression patterns of genes encoding photosynthesis-associated proteins and photoreceptors after dark treatment and re-exposure to white light. The differentially regulated genes include antenna proteins, components involved in oxidative photosynthesis and photoreceptors after dark treatment for 48 h (D48) and re-exposure to white light (WL) for 0.5, 6 and 24 h. Continuous white light (CWL) were control conditions. The color code indicates expression values, and squares with a diagonal line inside indicate genes with an expression ratio (log_2_ transformed) greater than +/−0.5 that are not significantly regulated. Genes where at least one of the two probes used for calculation of the expression ratios were significantly regulated by >2-fold at least at one time point during the experimental period were included in the figure. The expression patterns of genes marked with an asterisk indicate that they are chloroplast-encoded, and that the expression ratios was assessed by qRT-PCR instead of microarray analyses. The nuclear-encoded LHC48798 gene was not represented on the microarray, and were therefore also assessed by qRT-PCR analyses. The scale on the right represents gene expression ratio values, log_2_ transformed. The abbreviations used are LHCF: major fucoxanthin Chl a/c proteins; LHCR: red algal-like proteins; LHCX: LI818-like proteins; LHCZ: unknown function antenna protein, LHC#: unclassified light harvesting proteins, numbers refer to protein ID in JGI; LHL: Lhc-like; Psa: PSI proteins; Psb: PSII proteins; PETB-C: cytochrome b6f complex proteins; PETH: Ferredoxin-NADP reductase; PETJ: cytochrome c6; Atp: F-ATPase proteins; AUR: aureochrome; PAS: PER-ARNT-SIM domain, probably aureochrome; CRYL: cryptochrome-like protein; CPF: cryptochrome/photolyase family protein; SKP3: Sensor Kinase Protein 3.

The expression ratio of the differentially regulated (>2-fold at least at one time point during the experimental period) *P. tricornutum LHC* genes in D48-treated and re-illuminated cultures compared to CWL cultures are presented in Figure 5, and are grouped as suggested by the phylogenetic analysis. (The *LHCR9* and *LHC42519* genes were not regulated more than 2-fold at any time point and were therefore not included in the figure.) All genes encoding proteins belonging to the LHCY subclade, the LHCF group (except LHCF15 and LHC42519, the LHCR-I group and the deviant LHC17531 protein showed similar regulation patterns and could be classified as category 1 genes. They were all highly expressed in CWL, strongly down-regulated in D48 and started the climb back to CWL levels after re-illumination. The majority of these genes could be described by the three slightly different expression patterns described in Figure 2AI–III. The two patterns presented in Figure 2AI–II are common for several of the above-mentioned LHC genes and for many of the genes encoding enzymes necessary for production of Chl *a*. The third pattern illustrated in Figure 2AIII, was valid for several of the *LHCF* genes, and was distinguished from the two other patterns in that these genes never reached the CWL levels during the 24 h re-exposure time.

The *LHCF15* gene, which forms a distinct group with LHCF13 and LHCF14, exhibited, together with the *LHCX4* gene, a unique response to the treatments. These genes were both strongly up-regulated by the D48-treatment, and transcript levels did not return to CWL levels before the 24 h time point (Figure 5). Even though both genes showed a high expression in darkness compared to the CWL level, the *LHCF15* transcript was approximately 17 times more abundant than the *LHCX4* transcript, reaching a D48 expression level comparable to what is observed for the other *LHCF* genes when exposed to a prolonged period of WL. Whereas the *LHCX4* transcript level gradually decreased after re-exposure to light following the category 3 expression pattern (Figure 2C), the amount of *LHCF15* transcript stayed high after re-exposure to light for 0.5 h and 6 h, peaking at the 0.5 h time point.

The *LHCX1* transcript was, together with several of the *LHCF* transcripts, one of the most abundant mRNAs of the entire data set when exposed to light. Even though the *LHCX1* gene expression level was strongly down-regulated in darkness, the level of transcription was still several times higher than the average gene expression level of the data set. *LHCX1* reached expression levels close to CWL levels within 0.5 h of re-exposure to WL and stayed high throughout the 24 h re-illumination period. The *LHCX2* and *LHCX3* genes were strongly down-regulated in the dark and responded to re-illumination in a way similar to the *LHCR6-8* and *LHCR10* genes, even though they belong to different LHC subfamilies. The transcription of these genes were all strongly induced by 0.5 h exposure to WL after D48-treatment, while only showing a low to medium expression at other time-points, fitting the category 2 expression profile (Figure 2B). *LHCX2* displayed higher general expression levels than the other genes in this group, except at the 0.5 h re-exposure time point.

The *LHL1* expression ratio is also included in Figure 5. The transcriptional analyses of the *LHL1* gene suggest this gene to be a category 1 gene, displaying the expression profile illustrated in Figure 2AIII.

#### Photosystems and electron transport chain

The four multi-subunit complexes PSII, PSI, the cytochrome b6f complex and the F-ATPase are embedded in the thylakoid membrane of the chloroplast and catalyze oxygenic photosynthesis [37]. Whereas most genes encoding components of these complexes are localized to the chloroplast, all complexes except PSI (encoded by *Psa* genes) also contain one or several subunits encoded by nuclear genes. All nuclear-localized genes encoding subunits of PSII (*PSB* genes), cytochrome b6f complex (*PET* genes) and F-ATPase (*ATP* genes) were strongly suppressed by the D48-treatment, with a few exceptions. The *PSBP* gene showed a more modest down-regulation than the above-mentioned genes, and *PETC* was up-regulated in darkness (Figure 5). The nuclear-encoded *PETH* and *PETJ* genes, encoding Ferredoxin-NADP reductase and cytochrome c6 respectively, were also found to be strongly down-regulated by the D48-treatment. As described for other genes encoding components of the photosynthetic apparatus, the majority of the above-mentioned genes produced high-abundance transcripts in light, displayed the lowest expression in darkness and stabilized at or close to CWL levels after 24 h of re-illumination. The *PSBM*, *PSBO*, *PSBU*, *PSB31* and *PETJ* genes can all be classified as category 1 genes following the expression patterns schematically described in Figure 2_AII–III. The *PETC* and *PETC2* genes encoding the cytochrome b6-f complex iron-sulphur cluster subunit are located in adjacent positions (only 505 bp apart) on chromosome 11, and show 58% identity. Despite their chromosomal proximity, they displayed completely opposite transcriptional regulation. The *PETC* transcription level was in general low when the algae were exposed to light, but showed a 14-fold increase when the cells were D48-treated, following an expression pattern similar to the category 3 gene expression profile (Figure 2C). The *PETC2* gene, on the other hand, followed the more typical category 1 gene expression profile similar to the pattern depicted in Figure 2AI, only deviating from this pattern by reaching CWL levels already after 0.5 h re-exposure to light. The expression ratios of eight chloroplast genes (*PsaA, PsaE, PsbA, PsbV, PetB, PetD, AtpA, and AtpE*), encoding two subunits of each of the four above-mentioned complexes, were determined by qRT-PCR. In contrast to the nuclear-encoded components, the examined chloroplast genes showed a more moderate or no response to the D48-treatment, and were found to be highly expressed at all times (all Ct values ≤22), with *PsbA* exhibiting the highest expression of all. Six of the eight chloroplast genes displayed a log_2_ ratio of more than +/−1 at least at one time point, and are included in Figure 5 together with the ratios from the microarray analyses for the nuclear encoded subunits.

#### Photoreceptors

Photoreceptors can alter the biological activity of cells and organisms in response to light signals. *P. tricornutum* contains several putative blue-light receptors that belong to the family of aureochromes found in photosynthetic Stramenopiles (heterokonts) [38] and the widely distributed cryptochromes. Two putative phytochromes, which are red/far-red light receptors, have also been identified in the *P. tricornutum* genome. Expression ratios for the differentially regulated photoreceptor genes are presented in Figure 5. The transcriptional levels of all genes encoding the different photoreceptors, except *Diatom Phytochrome 1* (*DPH1*), were influenced by the D48-treatment and the subsequent re-exposure to light. Six out of nine photoreceptor gene transcripts showed higher expression levels in darkness than in CWL. Of these six genes, the putative phytochrome *SKP3* showed the category 3 expression profile schematically described in Figure 2C. The transcription of two aureochromes (*AUR3* and *PAS* (named *AUR2* in [39]) and *CPF2*, which encodes a cryptochrome/photolyase family protein, displayed lower or identical expression levels in darkness compared to CWL. *PAS* and *CPF2* transcription peaked after 0.5 h of re-illumination and displayed the category 2 expression profile depicted in Figure 2B. As for the majority of other genes described in the present study, the level of transcription from all genes encoding putative photoreceptors were close or back to CWL levels after 24 h of light-treatment.

### Cell Division in the Dark

The cell number was monitored throughout the first 35 h of the dark period, and was found to rise steadily the first 6 h (Figure S2). A 48% increase in cell number compared to CWL was observed before cell division ceased.

### Cell Pigment Ratios and Total Pigment Concentrations

**Figure 6:**
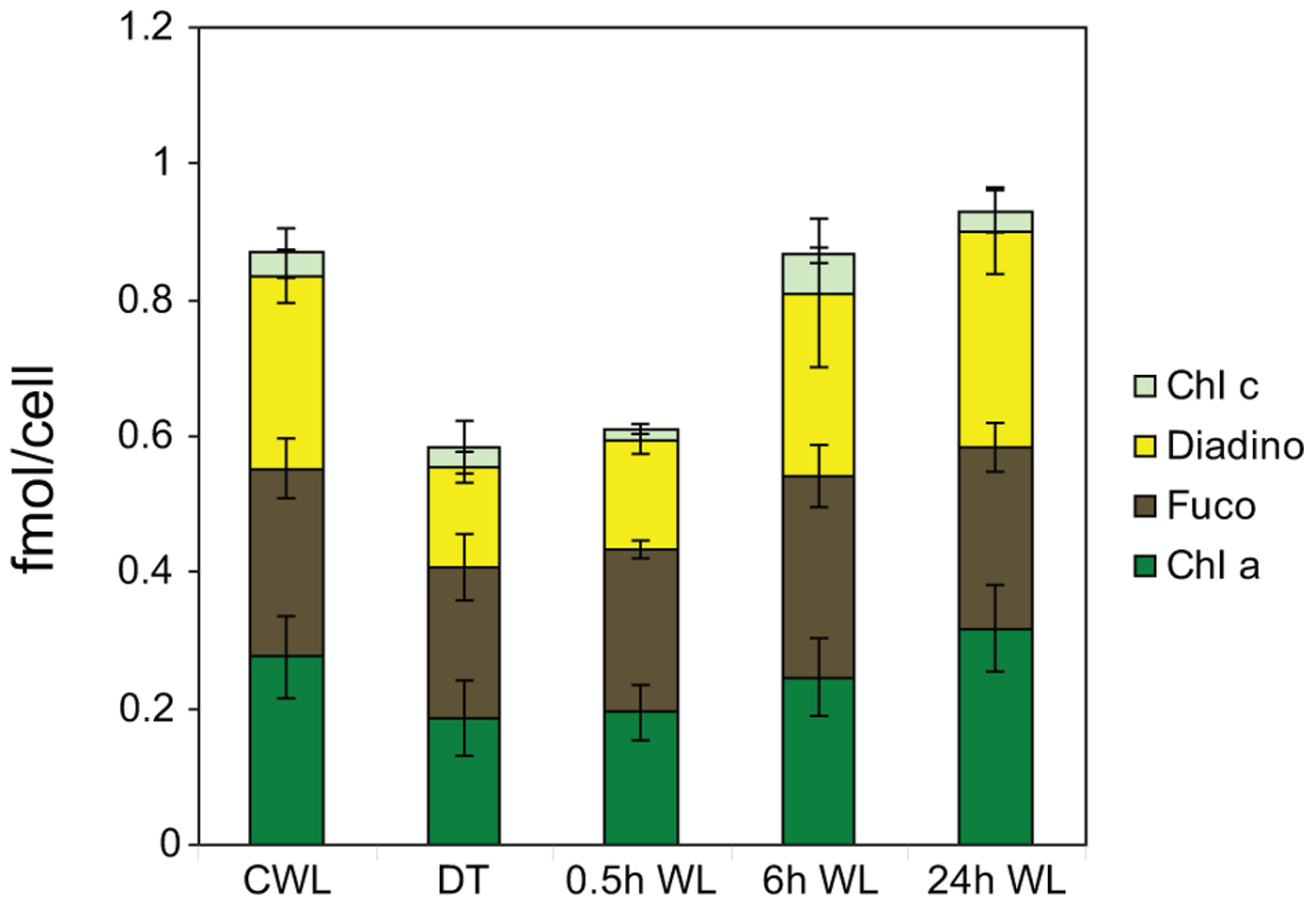
Changes in cell pigment concentrations after dark treatment and re-exposure to white light. Total cell pigment and cellular Chl *a*, Chl *c*, Fuco and Diadino concentrations (mol cell^−1^) for the samples from continuous white light (CWL), 48 h of darkness (D48) and 0.5, 6 and 24 h (WL 0.5–24 h) of re-exposure to CWL. The CWL and D48 sample values are a mean of 12 biological replicates. The WL 0.5–24 h values are the mean of three biological replicas. Values are averages of three parallel HPLC samples, and values are presented with ±SD bars.

The HPLC analysis showed that Chls *a* and *c (c_1_* and *c_2_)*, Fuco and Diadino were the dominating pigments in the algae. No Diato was detected in any of the five treatments. The fractions of Chl *a*, Chl *c* and Fuco to the total cell pigment concentration in each treatment were practically unchanged, while the fraction of Diadino was proportionally lower in D48 and 0.5 h WL-treated cells. The Diadino fraction did not reach the CWL value until 0.6 h of WL treatment. When left dark for 48 h, the total pigment concentration declined by 35% compared to CWL (Figure 6). Few changes in total pigment concentration had taken place 0.5 h after re-exposure to light. However, 6 h of re-exposure to light resulted in a sharp increase in the concentrations of all the individual pigments, almost reaching the CWL levels. No further increase in total pigment concentrations were observed at the 24 h time point.

### Variable Chl *a* Fluorescence

**Figure 7:**
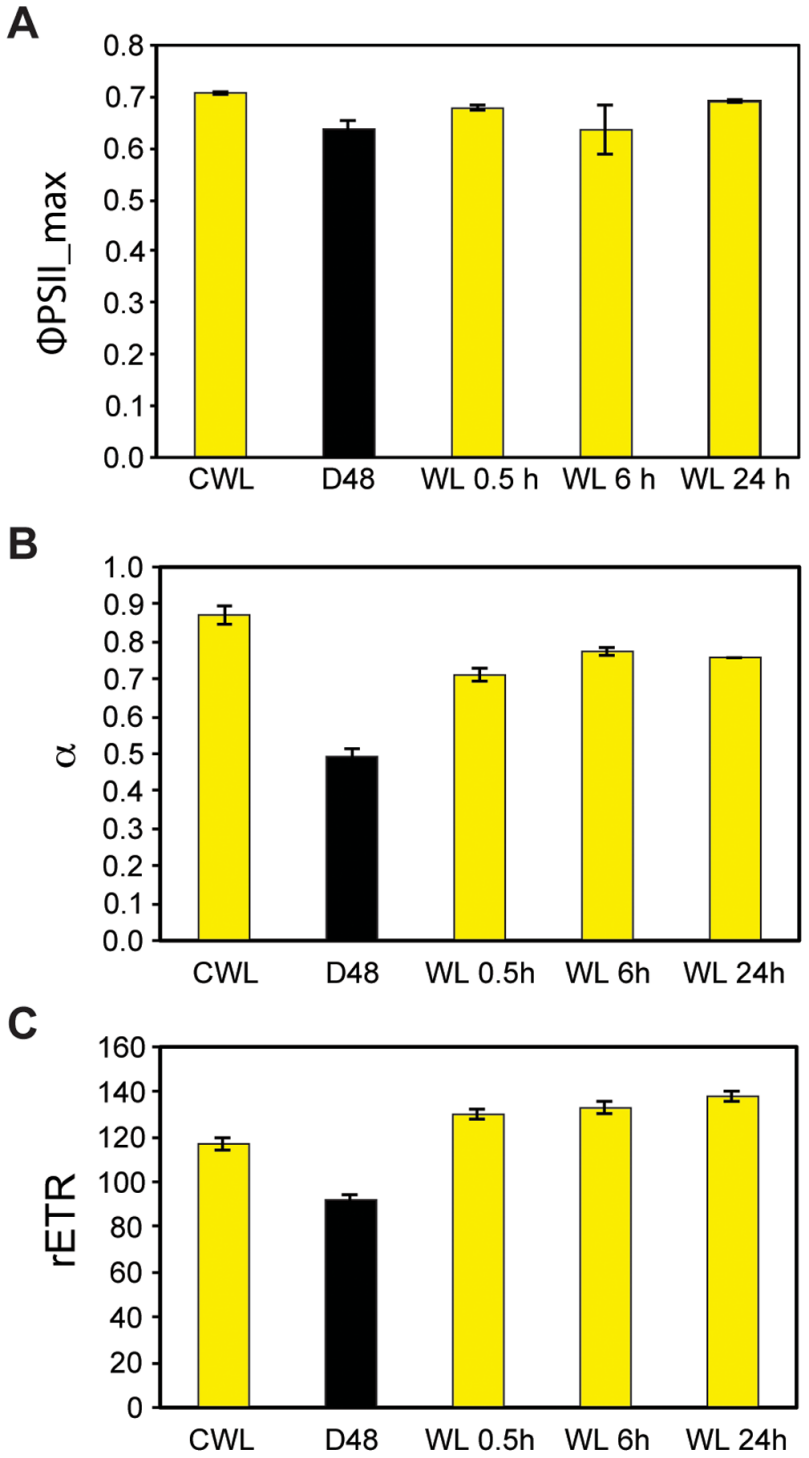
Photo-physiological dark-light responses by *in vivo* chlorophyll *a* fluorescence kinetics (PAM). A) Maximum quantum yield of charge separation in PSII (Φ_PSII_max_), B) maximum light utilization coefficient (α), and C) maximum rETR after exposure to continuous white light (CWL), 48 h of darkness (D48) and 0.5, 6 and 24 h of re-exposure to white light after darkness (WL 0.5–24 h). Φ_PSII_max_ was measured subsequently to a 3 min dark acclimation period and is a measure of the maximum efficiency of photosynthetic electron yield per mol photons absorbed in PSII. Bars are S.D. (n = 3).

The Chl *a* fluorescence kinetic signatures from PSII demonstrates the overall physiological response of *P. tricornutum* to the different treatments. It offers a good measure of the photosynthetic efficiency (maximum quantum yield of charge separation in PSII; Φ_PSII_max_), the photosynthetic capacity (maximum relative electron transport rate; rETR_max_) and the maximum light-utilization coefficient (the slope of the photosynthesis versus irradiance curve; α). Φ_PSII_max_ showed a 10% decrease in cells after D48-treatment compared to the CWL sample cells (Figure 7A). After 0.5 h of re-illumination, Φ_PSII_max_ increased approximately 7%, demonstrating an increase in the amount of electrons generated to photons absorbed in PSII. At 6 h of re-illumination the photosynthetic, Φ_PSII_max_ showed a slight drop before increasing again towards 24 h, where it almost reached the CWL value of 0.71. Both α (Figure 7B) and rETR_max_ (Figure 7C) were strongly reduced by the D48-treatment. After re-exposure to light, α increased quickly towards the level of the CWL sample, as measured after 0.5 h. The increase continued after 6 h and leveled off towards 24 h of re-illumination. Alfa never reached the initial level of the CWL sample during the course of this experiment. After 0.5 h of re-exposure to light, rETR_max_ increased, exceeding the capacity of the CWL cells, after which it leveled off much like α.

### *In vivo* Fluorescence Excitation Spectra

**Figure 8:**
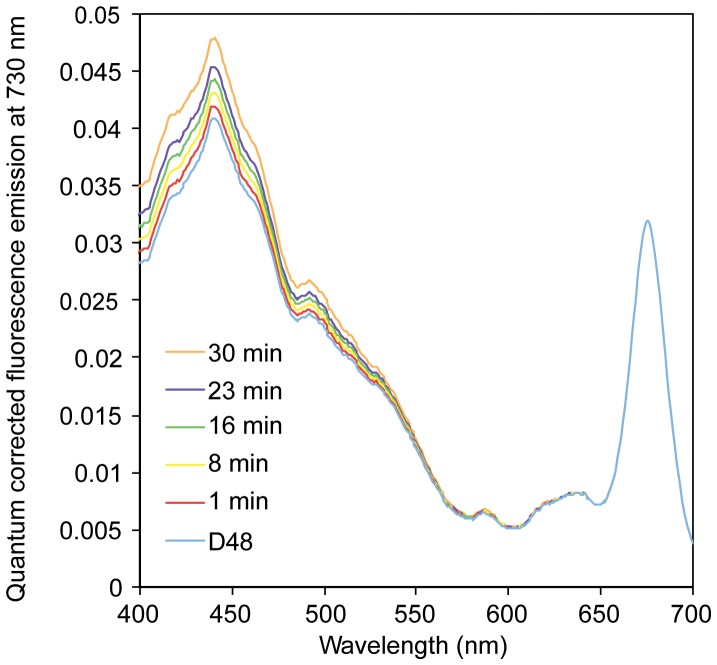
Changes in fluorescence emission during re-exposure of dark-treated cells. Time series of scaled quantum corrected fluorescence emission in *P. tricornutum* exposed to 48 h of darkness (D48) and subsequent 1–30 minutes of re-exposure to light. Fluorescence emission was measured at 730 nm to ensure origin from charge separation in reaction center II Chl *a* (P680).

*The in vivo* fluorescence excitation spectra indicate the relative light energy transfer efficiency of the different light-harvesting pigments to PSII reaction center Chl *a* within the PAR region (400–700 nm). Individual relative fluorescence excitation spectra were scaled to the Chl *a* fluorescence maximum at 675 nm in the D48 sample, and the subsequent 1–30 min of WL re-exposure of the same culture showed a gradual increase in light transfer efficiency between 400 and 535 nm from Chls *a* and *c* and Fuco to the PSII reaction center Chl *a* (Figure 8). At the *in vivo* excitation maximum of Chl *a* (∼440 nm), the increase was approximately 20% after 0.5 h of light exposure compared to the initial D48 value.

### Intracellular Structure

**Figure 9:**
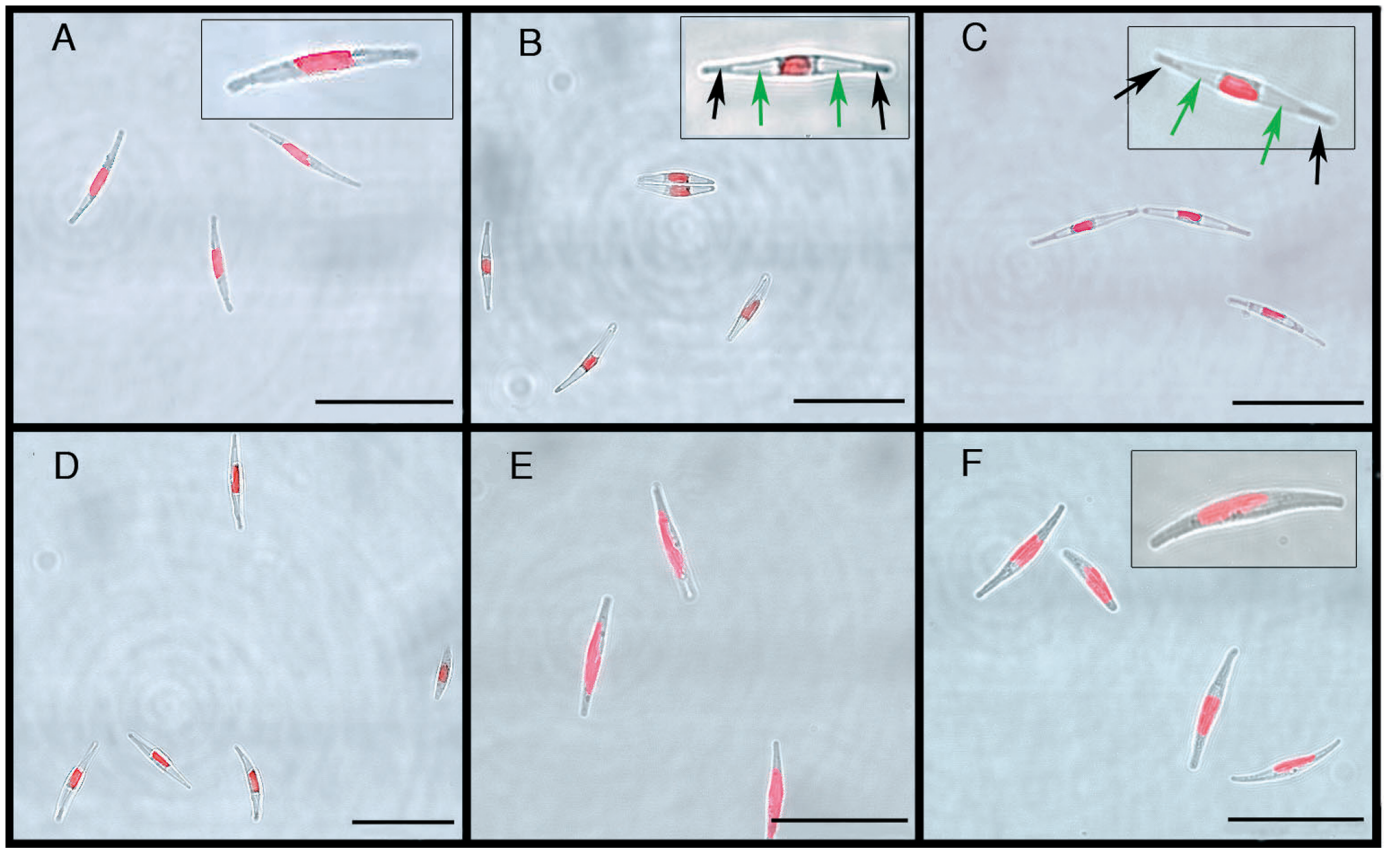
Intracellular changes during dark treatment. Intracellular differences in dark-treated *P. tricornutum* cells and cells re-exposed to moderate white light (E = 100 µmol m^−2^ s^−1^) of different duration. A) cells grown in continuous white light, B) cells exposed to 48 h of darkness (D48), C) cells exposed to 8 days of complete darkness, D–F) cells after white light (WL) re-exposure to 0.5, 6 and 24 h respectively. Inset in B and C: green arrows; vacuole-like structures, black arrows; cytoplasm. Scale bar, 25 µm.

Confocal light transmission images and Chl *a* epi-fluorescence images were combined to visualize changes in intracellular structure after prolonged D48-treatment and following re-exposure to WL (Figure 9). The *P. tricornutum* Bohlin clone Pt1 8.6 (CCMP632) has been described as a predominantly fusiform culture with cells containing one single, large golden-brown plastid in the central body [40], consistent with the appearance of cells grown at CWL (Figure 9A). Through electron microscope studies, Borowitzka and Volcani [41] identified vacuoles in the distal ends of exponentially growing fusiform *P. tricornutum* cells. These vacuoles were not visible in the CWL cells when studied with light microscopy, but in D48 cells, large vacuole-like structures occupied substantial parts of the cells (Figure 9B, green arrows in inset). The displaced cytoplasm could be seen as a grey structure in the tips of the cells (Figure 9B, black arrows in inset) and surrounding a contracted and rounded auto-fluorescent chloroplast. Further examination of cell cultures dark-treated for eight days (Figure 9C) confirmed the observations made after 48 h of darkness. Cells re-exposed to WL for 0.5 h appeared to have the same intracellular structure as the D48 samples (Figure 9D). In contrast, cells exposed to 6 and 24 h of re-illumination had regained normal intracellular appearance. The large vacuole-like structures had disappeared and the cytoplasm was expanded, filling the entire length of the cell. The chloroplast was bigger and elongated into the arms of the cell (Figure 9E–F).

## Discussion

In this study, we wanted to investigate the responses to prolonged darkness and the following acclimation to re-illumination in *P. tricornutum*. Previous reports hypothesize that the preservation of a functional photosynthetic apparatus during prolonged dark periods is an important survival mechanism enabling the diatoms to perform photosynthesis immediately after re-exposure to light [16], [17]. With this in mind, we have chosen to focus especially on pigments and genes encoding structural proteins that together comprise the light-harvesting antennae. We follow transcriptional profiles, pigment concentrations and profiles, morphological characteristics and physiological measurements to functionally analyze acclimation to light after a prolonged dark period.

### Gene Expression Categories

The transcriptional profiling indicates that the majority of the PhANGs and a few photoreceptor genes can be divided into three main categories based on their transcriptional responses to the dark-light treatments. The category 1 expression profile (Figure 2A) is found to be the most common light-responsive expression pattern for nuclear-localized genes (PhANGs) encoding enzymes and structural proteins needed in the formation and maintenance of a functional photosynthetic apparatus. This expression profile is recognized by high expression in CWL, low expression in darkness, a modest induction by 0.5 h re-exposure to WL and a rise towards CWL expression level during the following hours (WL 6 and 24 h). The category 2 expression profile (Figure 2B) is representative for the expression of several LHC genes belonging to LHC families thought to have a role in photoprotection rather than light harvesting [11], [28], [33], [42], [43], as well as for a few genes involved in the synthesis of pigments and two photoreceptor genes. The category 2 genes all display a strong response to re-illumination, peaking after 0.5 h of re-exposure to light. The Chl *a* pathway, the LHC family, the group of photoreceptors and oxidative photosynthesis components all contain a gene that can be described by the category 3 expression profile (Figure 2C). The transcripts from the category 3 genes are most abundant after prolonged darkness. When re-illuminated expression levels decrease gradually towards CWL levels.

### Transcriptional Profiles and Functional Roles of the *P. tricornutum* LHC Genes

The phylogenetic analyses of proteins belonging to the LHC superfamily revealed two main groups: one containing the original LHCF and LHCX clades, and the other containing the LHCR clade and a new clade called LHCY comprising three formerly unclassified *P. tricornutum* LHC proteins and LHCF16 (Figure 4). Organisms within the LHCY clade are so far restricted to the Haptophyceae, Bacillariophyta, Pelagophyceae, Phaeophyceae and Dinophyceae. The *P. tricornutum* LHC proteins annotated as LHCR1-14 were clearly divided into two separate subclades, with LHCR5-10 (LHCR-II) showing a closer relationship to a group of proteins with unknown function called the LHCZs [34] than to the rest of the LHCR proteins (LHCR-I). We identified a previously unclassified *P. tricornutum* protein (LHC15820) as a LHCZ protein and named it LHCZ1 based on its similarity to *Pavlova lutheri* Lhcz1. A recent phylogenetic analysis of LHCs from Chl *c*-containing algae [44] resulted in a division into seven clades that is similar to the one in this study. One exception is a small clade including PtLHC6062 that forms a sister clade to LHCR-I in [44] but is part of the LHCR-I clade in our study. The PtLHC6062 protein sequence used in this study is a corrected full-length version of the partial accession at GenBank, which may affect the phylogeny. Alternatively, reduced sampling of LHCs from non-diatom algae in our phylogeny may be the reason for this discrepancy.

The genes encoding proteins belonging to the LHCR-I, LHCY and LHCF (except LHCF15 and LHC42519) clades, and the gene encoding the deviant LHC17531 protein, display the category 1 expression profile (Figure 2A). Diurnal expression studies of “standard” fucoxanthin Chl *a/c* binding proteins in other diatoms [33], [45], [46] and Chl *a/b* proteins of green algae and higher plants [47] reports similar expression patterns of the “standard” *LHC* genes as those found in this study after transfer from darkness to light (Figure 2A and 5). *LHC* gene transcript levels are generally reported to be low in darkness, and display a gradual increase to reach a midday maximum after re-exposure to light. In Nymark et al. [28], these *LHC* genes were all found to be repressed by HL, which is the typical HL response displayed by *LHC* genes from both higher plants and algae encoding antenna proteins involved in light harvesting [33], [48], [49]. In contrast to our results, phylogenetic analyses performed by Dittami et al. [5] did not produce sufficient statistical support for the separation of the LHCFs and LHCRs, and the biological relevance of this division is questioned. The highly similar gene expression responses found for members of the LHCF, LHCR-I and LHCY groups to changes in light conditions in this and our previous HL study could indicate similar functional roles for the proteins encoded by these genes. However, in a recent study [11], different LHC proteins were found in the peripheral FCP complex of *P. tricornutum* compared to a FCP complex tightly bound to PSI. Of the above-mentioned LHC proteins, all proteins except LHCF12, LHCR4, LHCR13 and LHC6062 were identified in the peripheral FCP. In contrast, the FCP complex bound to PSI was enriched in LHCR proteins, containing several copies of each of the proteins in the LHCR-I group. LHCR proteins have been suggested to have a role in binding of Diadino cycle pigments. The only other LHC proteins associated with PSI were LHCF3/4 and LHC17531. Although both the *LHCF* and *LHCR-I* genes show similar regulation of expression, the study by Lepetit et al. [11] indicate that the LHCF and LHCR proteins play different roles in the light-harvesting complex and that the division into two groups is biologically relevant. The LHL1 protein of the RedCAP family was also found to be associated with PSI [50]. In contrast to other LHC-like proteins (e.g. early light-induced proteins (ELIPs), stress-enhanced proteins (SEPs), one helix proteins (OHPs)) that are generally associated with stress conditions [35], [51]–[53], the gene encoding the *P. tricornutum* LHL1 protein show no signs of being stress-induced when exposed to sudden changes in light conditions. The transcriptional response of the LHL1 gene is similar to the response of the LHCF and LHCR-1 genes both in the present experiment and in our previous HL experiment (LHL1 data not included in [28]).

The LHCR-II proteins were found to be closely related to a group of proteins with unknown function called the LHCZs [34], both in our analyses (Figure 4) and in the study performed by Dittami et al. [5]. LHCX2 and LHCX3 belongs to a different LHC subfamily than LHCR6-8 and LHCR10, but in our study the six genes encoding these proteins were all strongly but transiently induced by re-exposure to moderate light intensities for 0.5 h after D48-treatment (Figure 5), showing the category 2 expression profile (Figure 2B). In a previous study [28], we found that the same six genes were strongly induced by light stress, and that expression levels remained high for hours to days under HL conditions. Lepetit et al. [11] detected the LHCR6, LHCR8 and LHCR10 proteins in the peripheral *P. tricornutum* FCP complex only in HL cultured cells. They suggested that the role of these LHCR proteins might be binding of the increased amounts of Diadino cycle pigments in HL. Of the LHCX proteins, only LHCX1 was identified [11]. Several LHCX1 molecules were found in the peripheral FCP. The *P. tricornutum LHCX1* gene, encoding a protein crucial for non-photochemical quenching (NPQ) capacity in diatoms [42], was found to be constitutively expressed at very high levels at the moderate light intensity conditions used in this study. This is in accordance with results reported by Bailleul et al. [42], who reported that HL is not necessary for maximal *LHCX1* gene expression. The functional roles of the proteins encoded by the *LHCX2* and *LHCX3* genes and the LHCZ-related *LHCR-II* genes are not known, but several studies, including the present one, support a role for these genes in photoprotection rather than light harvesting [11], [28], [33], [42], [43].

In contrast to all the other LHC genes, *LHCF15* and the category 3 gene *LHCX4* both displayed high expression levels in darkness. The expression profiles of these genes were also divergent compared to the other LHC genes in our previous HL study. The protein products of these genes have not been detected in any of the FCP complexes in cells grown under WL of different intensities [11], [50], [54]. However, the LHCF15 protein level was recently found to be up-regulated in red light (RL) compared to blue light (BL), and suggested to be RL specific [55]. Despite the elevated expression levels of the *LHCX4* and *LHCF15* genes and also the LHCF15 protein [55] in response to specific experimental conditions, the functional roles of these genes remain unclear.

The proposed relationship between different *P. tricornutum* antenna proteins is supported by the gene transcription profiles obtained by the shifting dark-light treatment, suggesting that different functional roles can be assigned to the different subfamilies. The genes encoding members of the LHCF, LHCR-I and LHCY groups probably function in light harvesting, whereas the subgroups consisting of the proteins encoded by the LHCZ-related *LHCR-II* genes and the *LHCX* genes most likely play a role in light protection.

### Constant Cell Pigment Ratios

The ratios of the light-harvesting pigments to the total cell pigment concentration did not vary much between the five treatments (Figure 6). Considering the recent results pointing to an FCP monomer with fixed Chl *a*:Fuco:Chl *c*, this would explain the observed unchanged ratios of these pigments, as in previous works [11], [28], [56]–[58]. We also observed a 48% increase in cell number during the D48-treatment and a simultaneous 35% decrease in the cell pigment pool, which indicate re-distribution of already existing pigments to newly divided cells without *de novo* synthesis. Cells dividing in complete darkness would in this way have received a reduced number of “second-hand” photosystem core complexes and antennae structure proteins with fixed light-harvesting pigment ratios from their parent cell. In this experiment, a proportion of the Chl *a* was detected as Chl *a*-like pigments, which are not included in the presented pigment data, and would have increased the proportional ratio of Chl *a* to the other pigments. The Chl *a* not accounted for by the FCPs is bound by the core complexes of reaction center I and II [11] in a fixed, though uncertain number [59]–[64]. Diadino serves multiple purposes connected to both light harvesting and protection against photodamage, depending on the location within the FCP-PSI and FCP-PSII super-complexes [65]. The different localizations and heterogenic function of the Diadino cycle pigments explains their varying ratio to Chl *a*:Fuco:Chl *c* seen in this and other studies [11], [28], [56]–[58]. The cell pool of Diadino fell to a proportionally lower fraction in the D48 cells compared to the remaining pigments, before reaching the CWL fraction value after 6 h of light re-exposure (Figure 6). The 50% reduction in Diadino seen in the D48 cells might partly be explained by the mere 20% reduction in Fuco from CWL to D48. As Diadino is thought to serve as a reservoir for the synthesis of Fuco [12], this would be a convenient way of maintaining a high concentration of the main accessory light-harvesting pigment, despite the effect of pigment dilution and low transcript levels of genes encoding important enzymes necessary for carotenoid synthesis (Figure S1).

The absence of Diato might seem surprising, considering the sudden exposure of light after 48 h of complete darkness. In addition to its photoprotective role through NPQ in excess light conditions, this pigment is also usually detected as a result of chlororespiration in the dark [66]. Chlororespiratory energization of the thylakoid membrane is thought to maintain the ATP synthase in an active state in the dark, thereby facilitating ATP synthesis upon illumination [67]–[69]. The process of chlororespiration results in the reduction of the PQ pool by stromal reducing agents, which is coupled to acidification of the thylakoid lumen. This, in turn, activates the de-epoxidation reaction of Diadino to Diato [54] by violaxanthin de-epoxidase (VDE), also called diadinoxanthin de-epoxidase (DDE), even at a weak proton-gradient [68]. In *P. tricornutum* there is a constant increase in the reduction of the PQ-pool during dark-periods [68], that consequently would lead to an increasing accumulation of Diato in the dark. However, in most of the comparable dark-incubation experiments investigating chlororespiration and resulting xanthophyll cycling, the algae have been grown on a day:night growth regime [54], [66], [68]–[72]. This means that the algae would possess some degree of memory regarding the expectation of light. The *P. tricornutum* clone in this experiment, in contrast, has been grown at a 24 h light regime over several years, which means that it does not expect darkness. When left dark for 48 h, the cells display significantly reduced transcriptional activity, which is normally not observed during a normal night when grown on a day:night regime (Chauton et al. 2012, supplementary data). The lack of Diato in our dark-treated cells is at least partially explained by the very low expression of the *VDE/DDE* gene. This is supported by the study performed by Lavaud and co-workers [71], where the role of VDE in controlling NPQ was investigated by the generation of transformants of *P. tricornutum* in which the *VDE/DDE* gene was silenced. They concluded that the silencing of the *VDE/DDE* gene in *P. tricornutum* reduces Diato synthesis and NPQ.

### Effect of Gene Regulation at a Functional Level

Dark treatment of *P. tricornutum* cells leads to distinct intracellular changes, including contraction of the chloroplast and the appearance of large vacuole-like structures filling the arms of the cells. Diatom resting cells are generally characterized by densely granular and contracted cytoplasm and centered, rounded chloroplasts [73]. The latter was indeed the case in D48-treated *P. tricornutum* cells, whereas the reported changes of the cytoplasm common in resting cell formation [13]–[15], [18] were not observed even in cultures dark-treated for 8 days (Figure 9B–C). In spite of the apparent intracellular changes in D48-treated cells, typical resting cells do not seem to be produced during the length of this experiment. However, our results indicate that cells in this dark mode have a significantly lower nuclear transcriptional activity compared to cells in a “normal” state (Figure 1A, Table 1). In contrast, chloroplast-encoded transcripts are still present in high amounts, but these transcripts are probably not translated to proteins due to insufficient energy in the chloroplast, as observed in *Chlamydomonas* [74]–[76]. The gene expression data for category 1 genes (Figure 2A) suggest that there is very little or possibly no renewal of complexes containing nuclear-encoded subunits needed for performing photosynthesis when the cells are in dark mode. The cell pigment pool did not increase in the D48 cells, as is typically the case in LL-acclimated cells. On the contrary, it decreased by 1/3 from CWL to D48-treatment (Figure 6). The lower amounts of pigments in the D48-treated cells are in all likelihood a result of the pigment dilution effect caused by cell division. It is known that *P. tricornutum* cells arrest in the G_1_ phase after a period of prolonged darkness [20], [21]. During exponential growth *P. tricornutum* cells spend approximately 50% of their generation time in the G1 phase and 50% in S+G_2_+M [20]. When deprived of light, cells in the S, G_2_ or M phase complete the cycle before arresting in the G_1_ phase. This explains the ∼50% increase in cell number and decrease in cell pigment content during the 48 h dark period.

The Φ_PSII_max_ of the D48 cells showed a moderate drop compared to the CWL levels (Figure 7). This is not in accordance with the typically low Φ_PSII_max_ of LL acclimated algae [77], but the findings correspond to the observed decrease in cell pigment content (Figure 6). In addition, α decreased about 40% in the D48-treated cells compared to the CWL cells (Figure 7). This is also untypical for a LL acclimation response, where algae usually display a high α to yield a high photosynthetic efficiency while at the same time possessing low photosynthetic capacity [77]. However, a decrease of α with prolonged D48-treatment has previously also been observed in cyanobacteria isolated from cave systems absent of sunlight [78], [79]. In addition to the decreased cell pigment content, it is likely that the observed low α in the D48-treated cells is explained by reduced resonance energy transfer efficiency from the light-harvesting antenna pigments to the PSII reaction center, which is observed as changes in the fluorescence excitation spectra (Figure 8). Vernotte [79] speculated that the low α observed in dark-dwelling cyanobacteria is caused by structural changes within the light-harvesting antenna complexes, leading to inefficient resonance energy transfer between donor and acceptor pigment molecules.

Half an hour of re-illumination did not lead to a change in cell morphology, and the cells kept the dark mode appearance (Figure 9D). The nuclear transcriptional activity was higher than in darkness, but still lower than CWL levels (Figure 1A, Table 1). Re-illumination led to a weak induction of the category 1 genes, but the mRNA levels for these genes were still only a small fraction of the CWL levels (Figure 2A, Figure 3, Figure 5). In line with the weak response at the transcriptional level for several genes believed to be involved in pigment biosynthesis, pigment concentrations after 0.5 h light exposure were practically unchanged compared to the D48 cells (Figure 6). Despite of the very modest increase in photosynthesis-related transcripts and metabolites, the 0.5 h light treatment caused a steep increase in Φ_PSII_max_, α and rETR_max_ (Figure 7). The increases in the photosynthetic parameters correspond to an observed gradual increase in pigment resonance energy transfer efficiency between 400 and 535 nm (maximum absorption by Chls and Fuco) of 20% (Figure 8). The combined results suggest that the increase in photosynthetic parameters can be explained by a restructuring of PSII elements that were already present in the dark. Whether this delayed restructuring serves a light-protection purpose or is a process that simply requires light remains to be answered. A similar increase in pigment resonance energy transfer efficiency has previously been observed in cyanobacteria after prolonged dark-treatment [79]. In contrast to the low induction of category 1 genes (Figure 2A), 0.5 h of re-exposure to light strongly induced several LHC genes encoding proteins suspected to be involved in photoprotection (Figure 2B, Figure 5). Even though this transcriptional response implies that re-exposure to moderate light intensities after a prolonged dark period causes *P. tricornutum* cells to anticipate light stress conditions, well-known photoprotective mechanisms such as NPQ involving the conversion of Diadino to Diato was not detected.

Re-exposure to light for 6 h caused an elongation of the chloroplast and regaining of normal internal cell structure (Figure 9E). The results also imply that the nuclear transcriptional activity was back to pre-dark levels at this time point (Figure 1A, Table 1). The vast majority of genes involved in pigment formation and genes encoding the trans-membrane LHC proteins anchoring the pigments to the thylakoid membrane displayed high expression levels (Figure 2A, Figure 3, Figure 5) coinciding with the increasing pigment concentrations found at this time point (Figure 6). Dark treatment synchronizes the cells and re-illumination releases them synchronically [21]. Huysman and co-workers [21] reported that chloroplast division in *P. tricornutum* started 5 h after re-exposure to light following dark treatment. Although gene expression does not always correlate with protein expression, the observation stated above might explain the especially high levels of several gene transcripts involved in building of new antenna complexes detected at the 6 h time point, The Φ_PSII_max_ dropped slightly after 6 h, however, the standard deviation being almost an order of magnitude higher compared to 0.5 and 24 h (Figure 7A). In contrast, α and rETR_max_ continued to increase from 0.5 h to 6 h (Figure 7B and C), indicating that the algae were steadily improving their photosynthetic performance after being returned to the initial irradiance climate sustaining an efficient photosynthetic activity.

After 24 h of re-illumination, nuclear transcriptional activity, expression levels of PhANGs, pigment concentrations, intracellular structure, Φ_PSII_max_ and α were close to or back to CWL levels (Table 1, Figure 1–3, Figure 5–7, Figure 9). The rETR_max_ actually reached slightly higher levels after than prior to the D48-treatment (Figure 7C), suggesting that the cells not only regained their photosynthetic performance after 24 h of light re-exposure, but even showed an improvement when re-illuminated. The present study indicates that recovery of the photosynthetic machinery after D48-treatment seems to proceed in two phases, as suggested by Vernotte and co-workers [79]. The first phase involves a restructuring of PSII elements already existing in dark-treated cells (seconds to minutes), and the second requires protein and pigment synthesis (minutes to hours).

### Dark-light Shift Compared to LL-HL Shift

Both a shift from prolonged darkness to light and a shift from LL to HL involve an abrupt availability of a large amount of photons for light harvesting and photosynthesis. A comparison of the initial response (0.5 h) after a shift from prolonged darkness (D48) to WL (100 µmol m^−2^ s^−1^) compared to a shift from LL (35 µmol m^−2^ s^−1^) to HL (500 µmol m^−2^ s^−1^) [28] are presented in Table 2. Cells kept in complete darkness for a prolonged period of time perform no cell division, have lowered transcriptional activity and decreased cell pigment concentration, but keep the ability to perform photosynthesis intact. LL acclimated cells, on the other hand, are highly active dividing cells performing efficient photosynthesis by the use of a photosynthetic apparatus that contains high amounts of light-harvesting pigments [28]. Not surprisingly, with such different starting points, the initial response to increased light availability is quite different in the two situations (Table 1). Exposure to 0.5 h of light after a prolonged dark period weakly induces the transcription of genes encoding LHC proteins involved in light harvesting, components of PSII and enzymes involved in Chl *a* synthesis. In contrast, LL acclimated cells exposed to 0.5 h of HL display a general and in some cases a strong down-regulation of the same genes (Table 1, [28]). Several of the PhANGs mentioned above have been assigned to the category 1 group of genes in the present study. Even though the category 1 genes respond oppositely to the increased light availability in the two situations, they generally respond as a group in both studies, supporting the suggested classification. A common trait in both experiments is the strong induction of LHC genes thought to encode proteins that have a role in photoprotection, supporting the assignment of these genes to the category 2 group of genes.

**Table 2:** Initial responses to dark-light shift compared to light intensity shift.

		DT48-WL shift	LL-HL shift
**Molecular**	Global mRNA level	+	NC
	Genes encoding LHC proteins possibly involved in photoprotection	+++	+++
	Genes encoding LHC proteins involved in light harvesting.	+	÷
	Genes encoding components of PSII.	+	÷
	Genes encoding enzymes involved in Chl *a* biosynthesis	+	÷÷÷
**Metabolic**	Light harvesting pigments	NC	NC
	Photoprotective pigments: Diadino/Diato	NC/ND	÷/+
**Physiological**	Photosynthetic efficiency (Φ_PSII_max_)	+	÷
	Photosynthetic capacity (rETR_max_)	++	NC
	Maximum light utilization coefficient (α)	++	NC

Molecular, metabolic and physiological responses after a shift from prolonged darkness (D48) to white light (WL 0.5 h; 100 µmol m^−2^ s^−1^) compared to a shift from low light (LL 0.5 h; 35 µmol m^−2^ s^−1^) to high light (HL 0.5 h; 500 µmol m^−2^ s^−1^) [28].

Plus (+) and minus (÷) symbols indicate an increase or decrease in WL (0.5 h) compared to DT48, or HL (0.5 h) compared to LL levels. The abbreviations used are NC: no change; ND: not detected; LHC: light-harvesting complex; PSII: photosystem II; Chl: chlorophyll; Diadino: diadinoxanthin; Diato: Diatoxanthin; Φ_PSII_max_: maximum quantum yield of charge separation in PSII; rETR_max_: maximum relative electron transport rate; α: the slope of the photosynthesis versus irradiance curve.

The initial response to changes in light conditions does not include changes in the amount of light-harvesting pigments. Neither the up-regulation of genes encoding enzymes involved in Chl *a* synthesis after a dark-light shift, nor the strong down-regulation of the same genes after a LL-HL shift has any effect on the Chl *a* concentration at this early time point (Table 1, [28]). Whereas changes in light-harvesting pigment concentration is a relatively slow process (several hours), changes in pigment concentrations involved in a photoprotective response is a fast inducible process. In cells shifted from LL to HL conditions, a significant amount of Diadino is converted to Diato, thereby enabling NPQ to take place. In contrast, no Diato was detected after the dark-light shift. The moderate light intensity in this experiment was not high enough to require photoprotection by NPQ, and the 20% decrease in pigment resonance energy transfer observed in the D48 cells (Figure 8) might have contributed in protecting PSII reaction centers from the sudden light re-exposure during the first 30 minutes.

Φ_PSII_max_ displayed moderate changes after 0.5 h upon both the D48-WL and LL-HL shift. rETR_max_ and maximum light utilization coefficient (α) were practically unchanged after the LL to HL shift at this early time point, whereas these two parameters increased steeply after 0.5 h upon the D48-WL shift. LL to HL shifts involve a downsizing of the light-harvesting machinery, but the effect of the adjustments were not prominent at the physiological level before several hours into the HL acclimation period [28]. In cells experiencing a D48-WL shift, the suggested re-structuring of PSII elements already present in the D48 cells is likely to be a much faster process and might explain the rapid and steep increase in rETR_max_ and α after re-exposure to just 0.5 h of light (Figure 7).

### Conclusion

The shifts between CWL and prolonged darkness followed by the re-introduction of the diatom cells to the initial light climate led to strong responses at the transcriptional level. Gene expression patterns of members of the large gene family encoding light-harvesting complex proteins (LHCs) generally coincided with the division into different subfamilies, suggesting that different functional roles can be assigned to these subfamilies. The treatments also led to significant changes in pigment concentrations and intracellular morphology. These major changes only had a minor impact on the photosynthetic ability of the algae, which points to a photosynthetic apparatus in a functional stand-by status that enables immediate recovery upon re-illumination.

## Materials and Methods

The axenic *P. tricornutum* Bohlin clone Pt1 8.6 (CCMP632) culture was obtained from the culture collection of the Provasoli-Guillard National Center for Culture of Marine Phytoplankton, Bigelow Laboratory for Ocean Sciences, USA.

### Growth Conditions

Continuous culturing of the algae was performed as described in Nymark et al. [28]. The cultures were incubated at 15°C under cool white fluorescent light at a scalar irradiance (E_PAR_) of ∼100 µmol m^−2 ^s^−1^ under continuous white light (CWL) conditions. The cells divided 1.5 times per day under these conditions. Cell division continued 6 h after light deprivation, after which it ceased. The cultures were dark-treated for 48 h (D48) before re-exposure to the previous light conditions for 0.5 h, 6 h or 24 h (WL 0.5, 6 and 24 h). Sampling of CWL cultures and D48 cultures were performed in addition to the sampling at the WL incubation times of 0.5 h, 6 h, and 24 h. Three biological replicas for each of the five treatments were harvested to ensure statistical validation.

### Isolation of Total RNA and Processing

Total RNA was isolated from three biological replicas of diatoms cultured under CWL, D48 and subsequent re-exposure to WL 0.5 h, 6 h, or 24 h. Harvesting of diatom cultures and subsequent total RNA isolation, quantification and verification of RNA integrity were performed as described in Nymark et al. [28].

### DNA Microarray Experiments

Total RNA (150–200 ng) was reverse transcribed, amplified and labeled using the Low Input Quick Amp Labeling Kit, One-Color (Agilent p/n 5190–2305). 1650 ng cRNA from each sample was fragmented and hybridized on 4×44 K *P. tricornutum* whole-genome 60-mer oligonucleotide microarrays (Agilent Technologies) in an Agilent G2545A Hybridization rotary oven (10 rpm, 65°C, 17.5 h). Hybridization was performed with the Gene Expression Hybridization Kit (Agilent p/n 5188–5242). The slides were washed with buffer 1 & 2 from Gene Expression Wash Buffer kit (Agilent p/n 5188–5327) and scanned twice at 5 µm resolution on a laser scanner (G2505 B from Agilent Technologies), using the “dynamic range expander” option in the scanner software. The resulting images were processed using Agilent Feature Extraction software v9.5.

### Statistical Analyses of Microarray Data

The single color scan data (feature extraction files) were analyzed and genes with statistically significant differential expression were identified using the Limma package (version 3.2.3) [80] and R (version 2.10.1). Spots identified as feature outliers were excluded from analysis, and weak or non-detected spots were given reduced weight (0.5). The data were normalized using the quantile method, and no background subtraction was performed. A design matrix was created and contrasts between CWL against D48 and each of the re-exposure time points (WL 0.5 h, 6 h, or 24 h) were computed. The Benjamini and Hochberg's method was used to estimate the false discovery rate [81]. Genes with an adjusted p-value below 0.05 were considered to be statistically significant differentially expressed. The genes discussed in the text are represented by 2–5 different probes on each microarray. Presented expression ratios are an average of values obtained from the two probes closest to the 3′ end representing the genes in question. Supplementary information on significantly regulated *P. tricornutum* genes in this experiment are given in Table S1. The study is MIAME compliant. Raw data has been deposited in GEO (accession GSE 42039).

### Quantitative Real-time PCR

A two-step quantitative real-time PCR (qRT-PCR) was carried out on total RNA from the three biological replicas for each of the five treatments. The analysis was performed as described in Nymark et al. [28]. Forward and reverse primers used are listed in Table S2. LinRegPCR 11.1 software [82], [83] was used to determine the threshold cycle (Ct) value and PCR efficiency for each sample. The primer set efficiency was determined by calculating the mean of the efficiency values obtained from the individual samples. Relative expression ratios of eight chloroplast-encoded genes (*PsaA, PsaE, PsbA, PsbV, PetB, PetD, AtpA, and AtpE*) and five genes localized to the nucleus (*PSBP*, *PSB31, ATPC, LHCF15* and *LHC48798*) were calculated using the REST 2009 software [84]. The chloroplast-encoded target genes were normalized to a chloroplast-localized reference gene encoding Atp synthase cf1 beta chain (*atpB*; c35173-33746). A gene encoding dihydrolipoamide succinyltransferase related protein (*DLST*; Phatr2_45557) was used as reference gene to estimate relative expression ratios of the nuclear-encoded genes. The *DLST* gene is represented by five different probes on the microarray, and showed no response to the different treatments based on the microarray results. The primer efficiencies determined by LinRegPCR were included in the calculations. REST 2009 was also used to test significance of the expression ratio results of investigated transcripts by a Pair Wise Fixed Reallocation Randomization Test. Results of the qRT-PCR analyses of four out of five of the nuclear- encoded genes were only used to confirm the microarray results (data not shown). The *LHC48798* gene is not represented on the microarray, and relative expression ratios for this gene therefore had to be assessed by qRT-PCR analysis alone.

### Isolation of Polyadenylated mRNA

Poly(A) mRNA was isolated from total RNA from three biological replicas of cells from the five treatments. Isolation of mRNA was performed using the Oligotex mRNA kit (QIAGEN), which promises >90% recovery of poly(A) mRNA. 5 µg of total RNA from the 15 samples functioned as starting material for the procedure. The mRNA recovery was measured with a Qubit Fluorometer (Invitrogen), using the Quant-iT RNA assay kit (Invitrogen). The Student’s t-test was used to assess whether the total amount of mRNA in D48-treated samples and samples re-exposed to WL were significantly different from the amount found in CWL.

### Phylogenetic Analyses

LHC proteins in the NCBI (National Center for Biotechnology Information) protein database with highest homology to the *P. tricornutum* LHC proteins were selected for phylogenetic analyses. Accession numbers for the protein sequences used in the analysis are listed in Table S3. The LHC protein alignment was made with ClustalX (version 2.0) [85] and manually refined using GeneDoc [86]. A maximum-likelihood (ML) analysis of the protein alignment was performed using the RAxML program version 7.2.6 [87] with the PROTCATBLOSUM62 substitution model and running 100 bootstrap replicas. For visualization the best scoring ML tree was imported into Dendroscope (version 2.7.4) [88] and a circular cladogram was produced. IgLHC11 from the haptophyte *Isochrysis galbana* (Haptophyceae), which is related to the “deviant LHC” LHC17531, was selected as an outgroup.

### Pigment Analysis

The algal cultures were filtered gently for maximum one minute in 50 ml aliquots on GF/F glassfiber filters in the culture room at the given growth light regime and frozen immediately to ensure that the epo/de-epoxidation status in the cells were kept intact (Lohr and Wilhelm 1999). The filtering of the 48 h dark samples was performed in near darkness. Samples were kept at −20°C for one week before HPLC analysis. The HPLC pigment analysis was performed as described in Rodriguez et al. [89] using a Hewlett-Packard HPLC 1100 Series system. Twelve biological replicates each were used for the CWL and D48 samples. Three biological replicas each were used for the WL 0.5–24 h samples. The pigment values from the HPLC analysis were calculated as mol pigment per cell.

### Intracellular Structures

Micrographs of the different intracellular structures were taken by means of a Leica TCS-SL confocal laser scanning microscope with a 63× water objective. Two channels were used, one for non-confocal brightfield images of the entire algal cells and one for chloroplasts that were visualized by excitation with a 488 nm argon laser. Red Chl *a* autofluorescence (emission peak at ∼685 nm) from the chloroplasts was detected with a spectral detector set between 675 and 690 nm. The two channels were merged to identify the extension and location of the chloroplast within the algal cells.

### Variable *in vivo* Chl *a* Fluorescence (PAM)

Variable *in vivo* Chl *a* fluorescence was measured using the Pulse Amplitude Modulated (PAM) technique and a PhytoPAM (Walz, Germany) instrument. *In vivo* fluorescence was probed using a weak and non-actinic modulating LED light source (light emitting diode, Array-Cone PHYTO-ML, Walz, Germany), while light saturation of the photosystems was achieved using a strong red LED flash (>2000 µmol m^−2^ s^−1^, Actinic LED-Array-Cone). The latter ensured that all reaction centers of the photosystems were closed during the flash period. The instrument light source excites fluorescence at four different wavelengths; however, only fluorescence excitation at 665 nm was used. The nomenclature of van Kooten and Snell [90] was used, as also recommended by Suggett et al. [91]. The minimum (F_o_) and maximum fluorescence (F_m_) was measured at the end of a dark-acclimation period (3 min), and the maximum quantum yield of charge separation in PSII (Φ_PSII max_) was calculated as F_v_/F_m_, where F_v_ is the variable fluorescence emission (F_m_–F_o_). The photosynthesis *vs.* irradiance (P *vs.* E) relationship was measured by exposing the samples (after the 3 min dark-acclimation period) to 12 step-wise increasing irradiances (1 up to 1200 µmol photons m^−2^ s^−1^) at intervals of 30 s each. The operational quantum yield of PSII (Φ_PSII_) was calculated from the variable fluorescence (F_s_) at each irradiance and the maximum fluorescence measured after a saturation pulse (F_m_′) at the end of each irradiance intervals (F_m_′–F_s_)/F_m_′ [92], [93]. Maximum relative electron transport rates (rETR_max_) were calculated by multiplying Φ_PSII_ with the incubation irradiance. The relationship was fitted to determine the maximum light-utilization coefficient (α) and the maximum rETR using the ‘Webb equation’ [94] computed using least-squares criterion in SigmaPlot 10.0 (SYSTAT Software Inc., CA, USA). A Peltier cell (US-T/S, Walz) kept the temperature constant (±0.2°C) during incubations.

### *In vivo* Fluorescence Excitation

The fluorescence excitation spectrum of an alga at ambient temperature, results from the re-emission of light reaching chlorophyll Chl *a* of photosystem II (PSII) [95]–[97]. The fluoresced light has a longer wavelength than the absorbed light, and has two peaks in intensity at ∼680 [97], [98] and 730 nm [99]. For a fixed wavelength of emission, the intensity of fluorescence varies with the wavelength of incident light. This wavelength dependence is caused by the differential absorption of light by the pigment–protein complexes associated with PSII [100]. The photoprotective pigments are not included by this technique, as they absorb light but do not transmit the energy to any of the photosystems and hence do not contribute to the fluorescence [101], [102]. Fluorescence originating from PSI is negligibly small [97].

*In vivo* fluorescence excitation spectra, 400–700 nm were measured using a Hitachi F-3000 spectrofluorometer according to Johnsen and Sakshaug [103]. An infrared-transmitting glass filter (Schott RG 695 IR) in front of the photomultiplier (PMT), prevented visible direct and scattered light from the light source and cells by allowing only wavelengths >695 nm to pass. To ensure that no variable Chl *a* fluorescence was present, a time scan using excitation light at 440 nm and emission at 730 nm (1.5 min, same as scan time for fluorescence excitation spectrum) was recorded with DCMU (3-(3′,4′-dichlorophenyl)-1,1-dimethylurea)-treated cells (50 µM final concentration) before the fluorescence excitation spectra were recorded [89], [90]. The spectra were recorded at 1 nm spectral resolution (5 nm bandwidth), and the emission was monitored at 730 nm with 5 nm bandwidth [104]. The 730 nm emission peak of Chl *a* is used to avoid measuring both excitation and emission at ∼680 nm, which could destroy the light sensitive photomultiplier tube and cause interfering signals. For full visible range (400–700 nm) fluorescence excitation and for PAM, using the second emission peak of Chl *a* is widely accepted. The basic work behind the technique was performed by Neori et al. [104] who compared fluorescence excitation spectra with emission measured at both 685 nm and 730 nm with corresponding O_2_-action spectra. Both emission wavelengths gave similar results, except that the full visible range was not acquired using emission at 685 nm [89]–[91]. DCMU was added 1 minute prior to each individual measurement to avoid variable fluorescence, and spectra were recorded from a culture of D48-treated cells and from the same culture subsequently re-exposed to 1, 8, 16, 23 and 30 minutes of the initial 100 µmol m^−2 ^s^−1^ of WL. The fluorescence excitation measurements were quantum corrected using the dye Basic Blue 3 [105], [106], and the five light-exposure times were scaled to the red peak maximum of Chl *a* at 675 nm. This was done in order to be able study the development of light transfer efficiency by Chl *a*, Chl *c* and Fuco in the 400–500 nm range of the PAR spectrum, where they exhibit their maximum absorption [103], [107].

## Supporting Information

Figure S1 **Hypothesized carotenoid biosynthetic pathway in** ***P. tricornutum*** **modified from Dambek et al.** [**31**] **.** Colored squares indicate the regulation pattern of genes encoding putative enzymes involved in the synthesis of carotenoids after dark treatment for 48 h (D48) and re-exposure to WL for 0.5, 6 and 24 h. CWL was control conditions. The function of the carotenoid genes above the red line have been identified [31]. The function of the genes under the red line is still not established. Squares with a diagonal line inside indicate genes with an expression ratio (log_2_ transformed) greater than +/−0.5 that are not significantly regulated. The scale on the right represents gene expression ratio values, log_2_ transformed. The violaxanthin cycle (A) and the diadinoxanthin cycle (B) are boxed. The abbreviations used are: PSY, phytoene synthase; PDS, phytoene desaturase; ZDS, ζ-carotene desaturase, LCYB, lycopene β-cyclase; ZEP, zeaxanthin epoxidase; VDE, violaxanthin de de-epoxidase; VDL, violaxanthin de-epoxidase-like; VDR, violaxanthin de-epoxidase-related.
(EPS)

Figure S2 ***P. tricornutum*** **cell growth arrests in the dark.** Cell division in *P. tricornutum* culture from dark-incubation at time 0 and 35 hours into dark treatment.
(EPS)

Table S1 **Information on** ***P. tricornutum*** **genes discussed in the text.** Parameters given are the protein identification number (ID), chromosome location and NCBI accession numbers.
(XLS)

Table S2 **Primers used for quantitative real-time PCR.**
(PDF)

Table S3 **Protein sequences used for phylogenetic analysis of diatom LHCs.**
(PDF)
