# Supplementary material for: Molecular and Photosynthetic Responses to Prolonged Darkness and Subsequent Acclimation to Re-Illumination in the Diatom Phaeodactylum tricornutum
Source: PLoS One. 2013 Mar 8;8(3):e58722. doi: 10.1371/journal.pone.0058722 (PMC3592843; doi:10.1371/journal.pone.0058722)
Supplement: Table S2 — Primers used for quantitative real-time PCR. (PDF) [file pone.0058722.s004.pdf]

Gene	Accession	Orientation	Sequence	Amplicon size
PsaA	YP_874359	Forward	ATGCAGATGCTCATGACTTTGA	91
		Reverse	TACTGATAACTGTCCAAAGTGC	
PsaE	YP_874428	Forward	ATTGGTTTAACGAAGTCGGAAC	145
		Reverse	TGGACTGCTAACTTCAACTAAC	
PsbA	YP_874444	Forward	TGATATCGATGGTATTCGTGAG	101
		Reverse	TGGATACCGATAGCATTAGAAC	
PsbV	YP_874401	Forward	TTAATGCAACTTGTGGTGCTTG	121
		Reverse	AACCAAACCTGCTATGTTATCG	
PetB	YP_874393	Forward	GTTGTTGACGCATTTGCATCAG	98
		Reverse	CACCATCATACTTGCTGACCAA	
PetD	YP_874392	Forward	ATGGCAGCAGTTCCTTTAGGAT	148
		Reverse	GGTACACATGCACCTATACCTA	
AtpA	YP_874426	Forward	CGTATTGTGCAAGTTTGATGAC	65
		Reverse	TACGAACAATTTCGGTGTATGG	
AtpE	YP_874406	Forward	TCAAGTCGGGGTATTAGATGGT	141
		Reverse	AACAGTAACACGATCTCGATCA	
AtpB	YP_874407	Forward	ATTCAAGCTGTATACGTACCAG	70
		Reverse	TCTAAATGTGCGAATGTTGTCG	
ATPC	XP_002180505	Forward	TGGTCTCATTCAGCGACTAGGA	81
		Reverse	CAGCAGAGTGACCTTCTTGACT	
PSBP	XP_002182797	Forward	GCCTATCCTTCAGATGAAACGC	66
		Reverse	CCTGGAAAGCTTCTAGTAAGCC	
PSB31	XP_002180868	Forward	ATAAGGGAGACTTTGGCGCTGT	132
		Reverse	GCAGCAAAGATCTTGTTAGTGG	
LHCF15	XP_002183381	Forward	TTTGATGGCTTTGACAGACTCA	148
		Reverse	GTTCCTCATCTCCAAATAGGAT	
DLST	XP_002179864	Forward	CTTACAGAGAAGTTTGCCGAAG	148
		Reverse	TCATCTAATTCGTCAGAGCTGG	
