# Supplementary material for: Molecular and Photosynthetic Responses to Prolonged Darkness and Subsequent Acclimation to Re-Illumination in the Diatom Phaeodactylum tricornutum
Source: PLoS One. 2013 Mar 8;8(3):e58722. doi: 10.1371/journal.pone.0058722 (PMC3592843; doi:10.1371/journal.pone.0058722)
Supplement: Table S3 — Protein sequences used for phylogenetic analysis of diatom LHCs. (PDF) [file pone.0058722.s005.pdf]

Table S3. Protein sequences used for phylogenetic analysis of diatom LHCs.

Name	Accession	Organism
AaLHCR3	FC047617	Aureococcus anophagefferens
AaLHC39	EGB13073.1	Aureococcus anophagefferens
AaLHCF17	FC018279.1	Aureococcus anophagefferens
BnLHCZ1	DAA05895.1	Bigelowiella natans
CnLHCF2	EL620776.1	Chaetoceros neogracile
CnLHCF4	EL622142.1	Chaetoceros neogracile
CrLHCSR2	EDP01013.1	Chlamydomonas reinhardtii
CcFCP08	CAA04404.1	Cyclotella cryptica
CcFCP03	CAA04400.1	Cyclotella cryptica
CfFcpB-2	AAN08828.1	Cylindrotheca fusiformis
CfFcpB-1	AAN08827.1	Cylindrotheca fusiformis
EsLHCP34	CBJ32007.1	Ectocarpus siliculosus
EsLHCP30	CBJ30594.1	Ectocarpus siliculosus
EsLHCP4	CBN79580.1	Ectocarpus siliculosus
EsLHCP25	CBN78494.1	Ectocarpus siliculosus
EsLHCA2	CBJ31718.1	Ectocarpus siliculosus
EsLHCP14	CBJ27790.1	Ectocarpus siliculosus
EsLHCP32	CBJ31839.1	Ectocarpus siliculosus
EsLHCP8	CBJ26662.1	Ectocarpus siliculosus
EsLHCP23	CBJ28127.1	Ectocarpus siliculosus
EsLHCP42	CBJ33312.1	Ectocarpus siliculosus
EhLHCR1	Emihu1_442235 [a]	Emiliania huxleyi
EhLHCR2	Emihu1_451739 [a]	Emiliania huxleyi
EhLHCR4	Emihu1_439022 [a]	Emiliania huxleyi
Fracy1_187210	Fracy1_187210 [b]	Fragilariopsis cylindrus
Fracy1_270606	Fracy1_270606 [b]	Fragilariopsis cylindrus
Fracy1_208340	Fracy1_208340 [b]	Fragilariopsis cylindrus
Fracy1_261294	Fracy1_261294 [b]	Fragilariopsis cylindrus
Fracy1_269925	Fracy1_269925 [b]	Fragilariopsis cylindrus
Fracy1_209926	Fracy1_209926 [b]	Fragilariopsis cylindrus
Fracy1_270184	Fracy1_270184 [b]	Fragilariopsis cylindrus
Fracy1_269918	Fracy1_269918 [b]	Fragilariopsis cylindrus
Fracy1_213124	Fracy1_213124 [b]	Fragilariopsis cylindrus
Fracy1_273003	Fracy1_273003 [b]	Fragilariopsis cylindrus
Fracy1_186793	Fracy1_186793 [b]	Fragilariopsis cylindrus
Fracy1_218498	Fracy1_218498 [b]	Fragilariopsis cylindrus
Fracy1_272013	Fracy1_272013 [b]	Fragilariopsis cylindrus
Fracy1_272116	Fracy1_272116 [b]	Fragilariopsis cylindrus
Fracy1_269616	Fracy1_269616 [b]	Fragilariopsis cylindrus
Fracy1_169285	Fracy1_169285 [b]	Fragilariopsis cylindrus
Fracy1_172620	Fracy1_172620 [b]	Fragilariopsis cylindrus
Fracy1_175347	Fracy1_175347 [b]	Fragilariopsis cylindrus
Fracy1_211453	Fracy1_211453 [b]	Fragilariopsis cylindrus
Fracy1_273005	Fracy1_273005 [b]	Fragilariopsis cylindrus
Fracy1_272619	Fracy1_272619 [b]	Fragilariopsis cylindrus
Fracy1_268626	Fracy1_268626 [b]	Fragilariopsis cylindrus
Fracy1_210407	Fracy1_210407 [b]	Fragilariopsis cylindrus
Fracy1_271330	Fracy1_271330 [b]	Fragilariopsis cylindrus
Fracy1_272028	Fracy1_272028 [b]	Fragilariopsis cylindrus
GsLHCR4	CAC10533.1	Galdieria sulphuraria
GsLHCR2	CAC10535.1	Galdieria sulphuraria
GsLHCR3	CAC87419.1	Galdieria sulphuraria

GcLHCR3	DV968053.1	Gracilaria changii
GtLHC1	CAH25341.1	Guillardia theta
GtLHCP9	CAM33412.1	Guillardia theta
HtLHCR	AAW79364.1	Heterocapsa triquetra
IgLHCZ1	DAA05949.1	Isochrysis galbana
IgLHCP14	ABA55530.1	Isochrysis galbana
IgLHC5	ABA55521.1	Isochrysis galbana
IgLHC11	DQ118583.1	Isochrysis galbana
IgLHC15	ABA55531.1	Isochrysis galbana
MpFCPE	Q40301.1	Macrocystis pyrifera
PfLHCR3	FR738032.1	Pseudochattonella farcimen
PlLHCZ1	DAA05948.1	Pavlova lutheri
PlLHCF12	EC177330.1	Pavlova lutheri
PtLHCR9	XP_002186024.1	Phaeodactylum tricornutum
PtLHCR5	XP_002182761.1	Phaeodactylum tricornutum
PtLHCR6	XP_002181976.1	Phaeodactylum tricornutum
PtLHCR10	XP_002184869.1	Phaeodactylum tricornutum
PtLHCR8	XP_002176917.1.	Phaeodactylum tricornutum
PtLHCR7	XP_002177668.1	Phaeodactylum tricornutum
PtLHCR11	XP_002184127.1	Phaeodactylum tricornutum
PtLHCR4	XP_002177385.1	Phaeodactylum tricornutum
PtLHCR12	XP_002176857.1	Phaeodactylum tricornutum
PtLHCR13	XP_002182329.1	Phaeodactylum tricornutum
PtLHCR14	XP_002182162.1	Phaeodactylum tricornutum
PtLHCR1	XP_002178624.1	Phaeodactylum tricornutum
PtLHCR3	XP_002178019.1	Phaeodactylum tricornutum
PtLHCR2	XP_002183608.1	Phaeodactylum tricornutum
PtLHC_6062	XP_002182909.1	Phaeodactylum tricornutum
PtLHCZ1	XP_002183911.1	Phaeodactylum tricornutum
PtLHCX2	XP_002176987.1	Phaeodactylum tricornutum
PtLHCX1	XP_002179760.1	Phaeodactylum tricornutum
PtLHCX3	XP_002178699.1	Phaeodactylum tricornutum
PtLHCX4	XP_002182760.1	Phaeodactylum tricornutum
PtLHC24119	XP_002185437.1	Phaeodactylum tricornutum
PtLHCF16	XP_002178860.1	Phaeodactylum tricornutum
PtLHC48798	XP_002183454.1	Phaeodactylum tricornutum
PtLHC47485	XP_002181795.1	Phaeodactylum tricornutum
PtLHC17531	XP_002176735.1	Phaeodactylum tricornutum
PtLHCF13	XP_002183291.1	Phaeodactylum tricornutum
PtLHCF15	XP_002183381.1	Phaeodactylum tricornutum
PtLHCF14	XP_002186206.1	Phaeodactylum tricornutum
PtLHCF11	XP_002184619.1	Phaeodactylum tricornutum
PtLHCF5	XP_002184620.1	Phaeodactylum tricornutum
PtLHCF1	XP_002177871.1	Phaeodactylum tricornutum
PtLHCF2	XP_002177870.1	Phaeodactylum tricornutum
PtLHCF4	XP_002177868.1	Phaeodactylum tricornutum
PtLHCF3	EEC50683.1	Phaeodactylum tricornutum
PtLHCF8	XP_002182937.1	Phaeodactylum tricornutum
PtLHCF10	XP_002182219.1	Phaeodactylum tricornutum
PtLHCF6	XP_002182305.1	Phaeodactylum tricornutum
PtLHCF7	EEC43939.1	Phaeodactylum tricornutum
PtLHCF9	XP_002183709.1	Phaeodactylum tricornutum
PtLHCF12	XP_002184765.1	Phaeodactylum tricornutum
PtLHCF17	XP_002184763.1	Phaeodactylum tricornutum
PtLHC56448	XP_002177056.1	Phaeodactylum tricornutum

PpLHCX	EDQ67143.1	Physcomitrella patens subsp. patens
RhLhcc6	CAJ19158.1	Rhodomonas sp. CS24
SlLhcf4	AAG13005.1	Saccharina latissima
TpLHCR8	EED86590.1	Thalassiosira pseudonana
TpLHCR11	EED94694.1	Thalassiosira pseudonana
TpLHCR14	EED93079.1	Thalassiosira pseudonana
TpLHCR3	EED92402.1	Thalassiosira pseudonana
TpLHCA6	EED94441.1	Thalassiosira pseudonana
TpLHCA2	EED94820.1	Thalassiosira pseudonana
TpLHCZ1	AAFD02000008.1	Thalassiosira pseudonana
TpLHCX2	EED87488.1	Thalassiosira pseudonana
TpLHC270221	EED94157	Thalassiosira pseudonana
TpFCP_2	EED90181.1	Thalassiosira pseudonana
TpLHCR10	EED92032.1	Thalassiosira pseudonana
TpFCP3	EED87940.1	Thalassiosira pseudonana
TpLHCF7	EED93668.1	Thalassiosira pseudonana
TpLHCF11	EED95466.1	Thalassiosira pseudonana
TpLHCF2	EED88103.1	Thalassiosira pseudonana
TpLHCR5	ACI64366.1	Thalassiosira pseudonana
TpLHCR1	EED90328.1	Thalassiosira pseudonana
TpLhcx5	EED96716.1	Thalassiosira pseudonana
TpLhcx4	EED92507.1	Thalassiosira pseudonana
TpLhcx6	EED87487.1	Thalassiosira pseudonana
TpFCP2	EED93059.1	Thalassiosira pseudonana
UlLHCX	AJ891518.1	Ulva linza
UlLHCSR	ADY38581.1	Ulva linza
VcLHCX	EFJ50050.1	Volvox carteri f. nagariensis

[a]Gene ID from the Emiliania huxleyi genome assembly database (http://genome.jgi-psf.org/Emihu1/Emihu1.home.html)

[b]Gene ID from the Fragilariopsis cylindrus genome assembly database (http://genome.jgi-psf.org/Fracy1/Fracy1.home.html)
